# Stress Dissipation Encoded Silk Fibroin Electrode for the Athlete‐Beneficial Silk Bioelectronics

**DOI:** 10.1002/advs.202105420

**Published:** 2022-01-09

**Authors:** Woojin Choi, Deokjae Heo, Taeho Kim, Sungwon Jung, Moonhyun Choi, Jiwoong Heo, Jae‐Sung Kwon, Byeong‐Su Kim, Wonhwa Lee, Won‐Gun Koh, Jeong Ho Cho, Sangmin Lee, Jinkee Hong

**Affiliations:** ^1^ Department of Chemical and Biomolecular Engineering, College of Engineering Yonsei University Seoul 03722 Republic of Korea; ^2^ School of Mechanical Engineering Chung‐ang University 84, Heukseok‐ro, Dongjak‐gu Seoul 06974 Republic of Korea; ^3^ Department and Research Institute of Dental Biomaterials and Bioengineering and BK21 FOUR Project Yonsei University College of Dentistry Seoul 03722 Republic of Korea; ^4^ Department of Chemistry Yonsei University Seoul 03722 Republic of Korea; ^5^ Department of Chemistry Sungkyunkwan University Suwon 16419 Republic of Korea

**Keywords:** amino acid engineering, energy harvesting technology, intrinsic crosslink, mechanical property, silk fibroin, wearable bioelectronics

## Abstract

The kinetic body motions have guided the core‐shell fabrics of wearable bioelectronics to be elastoplastic. However, the polymeric electrodes follow the trade‐off relationship between toughness and stretchability. To this end, the stress dissipation encoded silk fibroin electrode is proposed as the core electrode of wearable bioelectronics. Significantly, the high degree of intrinsic stress dissipation is realized via an amino acid crosslink. The canonical phenolic amino acid (i.e., tyrosine) of silk fibroin is engineered to bridge the secondary structures. A sufficient crosslink network is constructed when tyrosine is exposed near the amorphous strand. The stress dissipative tyrosine crosslink affords 12.5‐fold increments of toughness (4.72 to 58.9 MJ m^−3^) and implements the elastoplastic silk fibroin. The harmony of elastoplastic core electrodes with shell fabrics enables the wearable bioelectronics to employ mechanical performance (elastoplasticity of 750 MJ m^−3^) and stable electrical response. The proposed wearable is capable of assisting the effective workouts via triboelectricity. In principle, active mobility with suggested wearables potentially relieves muscular fatigues and severe injuries during daily fitness.

## Graphical abstract

The stress dissipative network is encoded within the silk fibroin electrode via tyrosine crosslink. Since the phenol group of tyrosine links the secondary structures, the trade‐off relationship between toughness and stretchability has been overcome, meaning the realization of the elastoplasticity. Further, the elastoplastic silk fibroin electrode enables the energy harvesting wearables for the active athletes.

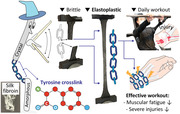

## 1 Introduction

The state‐of‐the‐art wearable bioelectronics has broadened toward routinely taking on/off garments or accessories, for example, smart clothes, watches, and spectacles.^[^ ^1^ ^]^ Especially, everyone irrespective of age and gender accompanies the wearables throughout their whole life. The clothes involve physiological factors, namely, aesthetic satisfaction and interpersonal relationships (e.g., uniform, T.P.O). Furthermore, the clothes could protect from the weather changes to disaster (e.g., flame, chemical, biological, radiological, and nuclear threats). Therefore, non‐skin‐tight and clothes‐types bioelectronics has been developed, such as optically communicable,^[^ ^2^ ^]^ energetically adaptive,^[^ ^3^ ^]^ or body signal responsive (temperature,^[^ ^4^ ^]^ sweat^[^ ^5^ ^]^) wearables.

The clothes‐type bioelectronics is comprised of core‐shell fibers and fabrics to realize user‐friendly functionalities.^[^ ^1^ ^]^ The core and shell correspond to the electrode and fabric, respectively. Since the routine body motions deform the components of wearables via ≈150% stretching and ≈90° torsion, the various polymeric electrodes have been proposed to alternate the solid metallic electrode.^[^ ^6^, ^7^ ^]^ Under the dynamic motions, in‐plane stress of the local intersection of warp and weft ranges from 150 MJ m^−3^ to 450 MJ m^−3^.^[^ ^8^, ^9^, ^10^ ^]^ Hence, the tough and stretchable, known as elastoplastic (toughness ≥ MJ m^−3^, maximum stress ≥ MPa, stretchability > 150%), electrode is essential in realizing futuristic bioelectronics.

However, the conformally on‐skin soft electrodes (e.g., 3D composite^[^ ^8^, ^11^ ^]^ and 1D fibrous electrodes^[^ ^12^ ^]^) hardly endure the motion‐induced stress due to the low toughness of < 100 MJ m^−3^. The softness (e.g., Young's modulus < 50 KPa^[^ ^13^ ^]^) is relevant to the linear relaxation of the skin bilayer (< 10%).^[^ ^14^, ^15^ ^]^ Furthermore, the structural property of polymeric electrodes limits the elastoplastic behavior. The polymers lose the stretchability of soft segments when the hard elements afford the toughness, meaning the trade‐off relationship of stretchability and toughness.^[^ ^16^, ^17^ ^]^ The innovative approach of electrode design is requisite to circumvent the inevitable limitation.

Hence, we have established the stress dissipation encoded biopolymer electrode to implement the elastoplastic electrode. Significantly, the intrinsic conformation was architectured as a crystal‐crosslinker‐amorphous strand. The crystal and amorphous regions are responsible for the toughness and stretchability, respectively.^[^ ^18^ ^]^ The amino acid crosslinker between secondary structures dissipated the applied stress and suppressed the fracture. The crosslinker was roled as the mechanical elements (e.g., spring and washer) of the versatile machines. The disulfide bond of cysteine, representative of protein crosslink, manipulates the peptide assembly. Due to the limited redox sensitivity of the disulfide linkage, the various approaches (e.g., chelation,^[^ ^19^ ^]^ amine‐specific covalent bond,^[^ ^20^ ^]^ and noncanonical amino acid^[^ ^21^ ^]^) have been investigated to connect internal protein strands. Furthermore, the technological fusion (genetic anchoring of photo‐crosslinkers^[^ ^22^ ^]^) produced the advanced crosslink. However, the phased‐in reagents increase the systemic degree of freedom due to the element diversity. Furthermore, the cytotoxicity of crosslinkers limited the production of user‐friendly bioelectronics.

To this end, the side group of canonical amino acid is proposed as the inherent crosslinker to scatter the mechanical load. Specifically, tyrosine was regarded as the model crosslinker. The phenolic (i.e., aromatic and polar) group could form the covalent and hydrogen bonding with adjacent protein structures.^[^ ^23^ ^]^ For instance, the wing tendons of dragonflies present structural stability due to the tyrosine crosslink within resilin.^[^ ^24^ ^]^ Besides, the insect‐derived silk fibroin was adopted as the model biopolymer. In nature, the tyrosine (≈ 5.4 mol% in *B. Mori* silk fibroin) is the linker sequence within the silk fibroin strand, which interconnects the flanking crystal and amorphous domains.^[^ ^23^ ^]^ The encoded tyrosine crosslink dissipated the applied stress, enhanced the toughness ≈12.5 times, and realized the elastoplastic silk fibroin electrode (EPSF).

**Figure 1:**
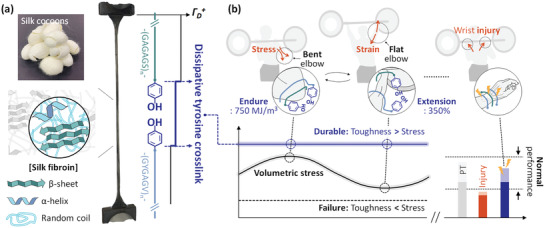
Illustration of the elastoplastic electrode and corresponding bioelectronics. a) The source of silk fibroin is the silk cocoons from *B. Mori*. The heavy chain of ‐(GAGAGS)_n_‐ and light chain of ‐(GYGAGV)_n_‐ are depicted in green and blue, respectively. The secondary structures of chains (*β*‐sheet, *α*‐helix, and random coil) are described. The phenolic side group of tyrosine is highlighted in navy color. The tyrosine crosslink increases the degree of stress dissipation (*Γ*_D_). b) During the daily workout (e.g., military press), the fabric components of wearable bioelectronics are exposed to various deformations such as volumetric stress or stretching. The principal parameter for sustainable bioelectronics is the outstanding toughness over the motion‐induced stress (≈750 MJ m^−3^) and sufficient stretchability (≈350%). The proposed bioelectronics could support the athlete employing triboelectricity. Ideally, the athlete could keep the regular performance alone without injuries.

In this study, EPSF was produced as the core electrode of wearable bioelectronics. Then, the core‐shell EPSF fabric band and prototype of EPSF bioelectronics were further proposed. Thanks to the effective stress dissipation from the core electrode, the EPSF fabric band was able to overcome the motion‐induced volumetric stress (**Figure** **1**a). Furthermore, the harmony of core EPSF with the shell fabrics (e.g., Gore‐tex and nylon) featured the super‐elastoplasticity of 750 MJ m^−3^ toughness and 350% stretchability (Figure 1b). The prototype of EPSF bioelectronics supported the active workout efficiency in the analogous methods with personal trainers.^[^ ^25^ ^]^ In principle, the widespread muscle fatigue (e.g., myalgia, soreness) may be potentially suppressed,^[^ ^26^ ^]^ and the standard posture can be coached exploiting the triboelectric energy generated during workouts (Figure 1b). In contrast, traditional smart clothes sense less beneficial information such as electrocardiograms and body temperature.^[^ ^27^ ^]^ Hence, the next‐generative functions above are positioned at the heart of the athletes’ demands.

## 2 Results and Discussion

### 2.1 Investigation of Silk Fibroin Electrodes in Terms of *β*‐Sheet Crystallites

Both secondary structures and tyrosine crosslinks are highly influential for the mechanical properties of silk fibroin electrodes. For instance, the researchers engineered the resilient *β*‐sheet crystals to implement the epidermal electronics.^[^ ^30^ ^]^ On the other hand, the intrinsic tyrosine crosslink is a vital driving force in the structural stabilization of insects; for instance, dragonfly and silk fibroin.^[^ ^24^, ^31^ ^]^ To determine that only tyrosine crosslink influenced the mechanics of silk fibroin electrodes, the various silk fibroin electrodes were designed to be comprised of analogous secondary structures. *β*‐sheet crystals are embedded in the less‐ordered (semi‐amorphous) matrix (Figure 1a).^[^ ^18^ ^]^ Due to the molecular packing of heavy chains, *β*‐sheet crystals are the principal component for outstanding toughness. For instance, the internal stiffness was 600 times higher in the crystal than in the amorphous region (576 pN Å^−1^ of crystal and 9.9 pN Å^−1^ of amorphous strand).^[^ ^28^ ^]^ The crystal transiently endures the transferred load; however, it fails directly without sufficient unraveling.^[^ ^32^, ^33^ ^]^ On the other hand, the amorphous strand could be linearly elongated ≈150% under the weak tensile force.^[^ ^34^ ^]^ Hence, the crystal and amorphous strand are responsible for the toughness and stretchability of the silk fibroin electrode.

**Figure 2:**
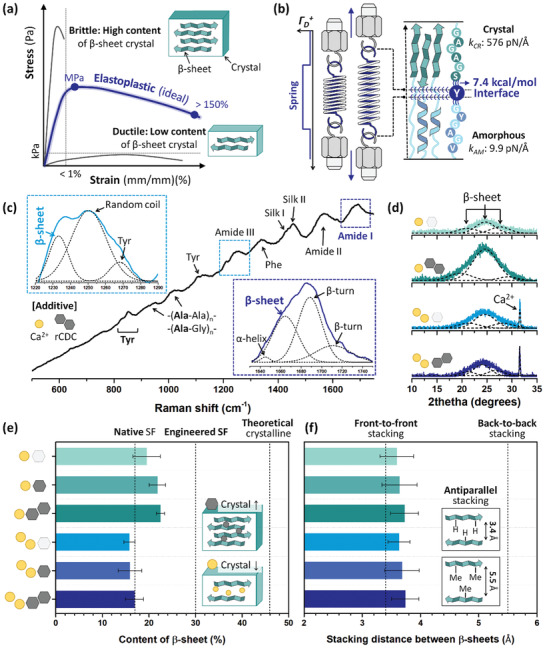
*β*‐sheet crystal‐related properties of silk fibroin electrodes. a) Representative strain versus stress curve with different contents of *β*‐sheet crystal. Dense distribution of crystals results in the brittleness (i.e., maximum stress > MPa, fracture strain < 1%). However, the least crystal content is responsible for the ductile silk fibroin electrode (i.e., maximum stress < kPa, fracture strain > 200%). The EPSF (i.e., maximum stress ≥ MPa, fracture strain > 150%) is desirable for the core electrode of the wearable bioelectronics. b) Illustration of stress dissipative tyrosine crosslink (Tyr; Y) inspired from the mechanical element (e.g., spring). The values of 576 and 9.9 pN Å^−1^ represent the stiffness of the unit crystal and amorphous, respectively.^[^ ^28^ ^]^ 7.4 kcal mol^−1^ indicates the crosslink strength exerted by tyrosine.^[^ ^29^ ^]^ c–f) Parametric studies about *β*‐sheet crystal within various conditions of silk fibroin electrodes. Yellow circle (○) and black hexagon (⬡) indicates the divalent cation (Ca^2+^) and 2D carbon, respectively. (c) Representative Raman spectrum of silk fibroin electrode with ○ = 6 wt.% and ⬡ = 1 wt.%. (d) X‐ray diffraction patterns of silk fibroin electrode with ○ = 3, 6 wt.% and ⬡ = 0, 1 wt.%. (e) Contents of *β*‐sheet crystal and (f) stacking distances between *β*‐sheets of silk fibroin electrodes with different additive concentrations. The dashed lines indicate the characteristic values of well‐investigated silk fibroins.

Since the limited number of canonical amino acids (*i.e.*, Gly ≈ 45%, Ala ≈ 25%, Ser ≈ 12%, Tyr ≈ 5%) comprise the secondary structures, the relative content of crystal and amorphous strand features the trade‐off relationship. In other words, the stretchability of silk fibroin inevitably reduces when the toughness increases according to the high content of *β*‐sheet crystal. For instance, the methanol‐treated *β*‐sheet rich silk fibroin (crystal content > 30%) showed the brittle material behavior; that is, the superior maximum strength of ≈60 MPa with a low breaking strain of ≈2.1%.^[^ ^18^ ^]^ However, *β*‐sheet poor silk fibroin (< 15%) presented the tensile response of ductile material; for instance, the low maximum strength of < 50 kPa with the superior stretchability of > 200%.^[^ ^29^ ^]^ Hence, the realization of EPSF is challenging due to the intrinsic trade‐off relationship of secondary structure‐related mechanical properties (**Figure** **2**a).

Inspired from the spring and washer to improve the mechanical performance (Figure S1, Supporting Information), the tyrosine is engineered as the stress dissipative crosslinker to implement the EPSF (Figure 2b). The tyrosine features the rigid hydrogen bonding with the surrounding building blocks; that is, 4.6, 4.7, and 7.4 kcal mol^−1^ versus ‐C═O, ‐OH, and ‐NH, respectively.^[^ ^29^ ^]^ Besides, the tyrosine crosslink network could consume the applied stress through the anisotropic rotation and suppress the stress propagation.^[^ ^35^ ^]^

Here, two additives (i.e., Ca^2+^, 2D carbon) were introduced to fabricate various silk fibroin electrodes with comparable *β*‐sheet crystal properties and different degrees of tyrosine crosslink. In other words, the role of *β*‐sheet crystal (i.e., content and size effect) was screened out from the principal parameter (Figure 2c–f). Hence, the stress dissipation from tyrosine crosslink was solely responsible for elastoplastic behavior. In terms of *β*‐sheet crystallites, two additives presented the complementary influences. Divalent cation impedes the interchain energy between GAGAGA sequences from −180.6 to −176.2 kJ mol^−1^. Thus, Ca^2+^ inhibits the packing of *β*‐sheets and reduces the crystal content.^[^ ^36^ ^]^ On the other hand, 2D carbons facilitate the assembly of amino acids and enhance the crystal content.^[^ ^29^ ^]^ Since 2D carbon was originated from the polysaccharide comprising of the acetamide group and glucosamine ring, 2D carbon is highly nitrogen‐doped (≈25.5%) (Figure S2, Supporting Information). Therefore, the mild concentration of 2D carbon (≈1 wt%) was influential in crystallization due to the interfacial interactions of nitrogen groups with hydrophilic moieties of silk fibroin.^[^ ^37^, ^38^ ^]^

According to Raman spectroscopy, the various vibrations of building blocks (i.e., Gly, Ala, Tyr) were monitored (Figure 2c). The dominant building blocks were double‐checked via Fourier‐transform infrared spectroscopy (Figure S3, Supporting Information). The relative intensity within ≈1400–1500 cm^−1^ indicated that the prepared silk fibroin electrodes were composed of stable silk II conformation regardless of additive concentrations (Figure S4, Supporting Information). The vibrations of C═O isotopes (amide I; ≈1630–1750 cm^−1^ and amide III; ≈1220–1290 cm^−1^) informed the secondary structures. X‐ray diffractions of electrodes presented *β*‐sheet‐related peaks at 2*θ* of ≈21.1°, ≈24.5°, and ≈27.6° (Figure 2d). The intense peak at ≈24.5° is originated from the polar arrangement of *β*‐sheet stacking.^[^ ^18^, ^39^ ^]^ Besides, Ca^2+^ peaks (2*θ* ≈ 31.6°) occurred as the concentration of cation increased.

The diverse technologies realized the manipulation of *β*‐sheet contents within the range of native (≈18%) and theoretically calculated (≈47%) values.^[^ ^18^ ^]^ Especially, the dramatic variations of crystallization (Δ_max_ > 15%) resulted in the distinguishable transition of mechanical responses.^[^ ^29^ ^]^ Regarding the well‐studied Raman scattering of amide I vibration, the contents of *β*‐sheet were quantified as a function of additive conditions (Figure 2e). The observed crystal contents followed the findings of prior studies. 2D carbon upregulated the crystallization;^[^ ^29^ ^]^ however, Ca^2+^ hindered *β*‐sheet stacking.^[^ ^36^ ^]^ Thanks to the complementary additive chemistry, the contents of the *β*‐sheet presented a narrow variation range within empirical values; that is, ≈16.9% to ≈22.5% (Δ_max_ ≈ 5.6%).

The size effect of *β*‐sheet crystals (i.e., nanoconfinement) controls the mechanics of silk fibroin.^[^ ^40^ ^]^ For instance, 10% increase of hydrogen bonding distance could determine the secondary structures (*β*‐sheet crystal or *β*‐turn amorphous strand).^[^ ^18^ ^]^ The stacking distances between *β*‐sheets were consistent in the range of the values of small amino acids (i.e., Gly, Ala) (Figure 2f). The additives might intercalate into the *β*‐sheet crystals and marginally variate the stacking distance from ≈3.68 to ≈3.73 Å (Δ_max_ ≈ 1%). Especially, stacking distances suggested that front(Gly)‐to‐front stacking is the principal driving force rather than back(Ala)‐to‐back interaction. Furthermore, the vibrational spectra indicated that the poly(Ala) was abundant within silk fibroin electrodes (Figure 2c and Figure S3, Supporting Information). Hence, the *β*‐sheet crystal conditions of various silk fibroin electrodes were identical, suggesting the effect of *β*‐sheet crystal in mechanics was the minor parameter.

### 2.2 Stress Dissipative Tyrosine Crosslink Enabled the Elastoplastic Silk Fibroin Electrode

**Figure 3:**
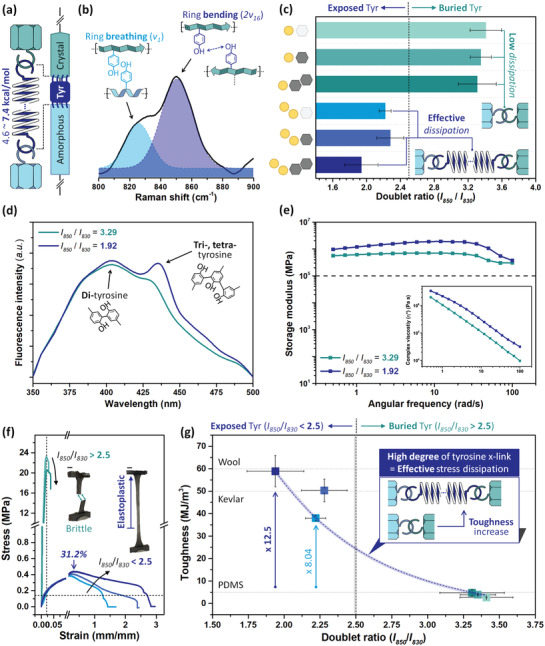
Characterization of stress dissipation encoded silk fibroin electrode. a) Illustration of the intrinsic structure of EPSF. Here, the tyrosine crosslink corresponds to the spring of hook connection. b) Representative Raman spectrum (≈800–900 cm^−1^) informing the degree of tyrosine crosslink. c) The doublet ratios (*I*_850_/*I*_830_) of silk fibroin electrodes were parameterized. The efficiency of stress dissipation within the silk fibroin electrode is depicted as a hook connection. d) Fluorescence emission spectra and e) rheological responses of silk fibroin electrode with representative doublet ratios; that is, *I*_850_/*I*_830_ = 1.92 and 3.29. f) Engineering strain versus stress curves of silk fibroin electrode with various doublet ratios. The scale bar of insert images is 1.0 cm. g) Summarized toughness of silk fibroin electrodes as a function of doublet ratio. The horizontal dashed lines indicate the toughness of the wool, Kevlar, and PDMS.

The stress dissipation through the crosslinked network is essential in fatigue resistance and mechanical performance.^[^ ^16^, ^17^ ^]^ As shown in Equation (1), the stress dissipation (*Γ*_D_) is linearly proportional to the material toughness (*Γ*_T_); thus, it is essential in realizing the EPSF.^[^ ^41^, ^42^ ^]^

(1) $$\;\Gamma_{T}=\Gamma_{o}\;+\Gamma_{D}\;$$

where the subscripts T, O, and D indicate the total, internal, and dissipative toughness, respectively. Hence, the crystal‐crosslinker‐amorphous conformation was proposed as the internal architecture of the EPSF (**Figure** **3**a). According to the coarse‐grained approach, the unit amino acid was responsible for the macroscopic performance.^[^ ^28^, ^43^, ^44^ ^]^ For instance, the average chain modulus (*E*_c_) is decisive on the systemic modulus (*E*), as shown in Equation (2).^[^ ^45^ ^]^

(2) $$\;\frac{1}{E}=\frac{1}{E_{c}}\;+\frac{\left\langle \mathrm{si}n^{2}\theta \right\rangle }{G}$$

where *G* denotes the shear modulus; <sin^2^ *θ*> represents the mean orientation of the unit chains.

Significantly, the phenolic amino acid tyrosine was contemplated as the crosslinker of the EPSF. The tyrosine content is especially abundant in *Bombyx mori* silk fibroin (≈5.4 mol%); that is, ≈5 times plentiful compared to the other natural biopolymers, such as 1.20 mol% of human hair keratin.^[^ ^46^ ^]^ The aromatic and polar functional groups interlocked the interface of dissimilar domains via hydrogen bondings. The hydrogen‐bonded matrix can dissipate the applied mechanical stress. The theoretical scenarios of in situ crack propagation evidenced the significant role of tyrosine crosslink (Figure S5, Supporting Information). The amorphous side crack tip underwent approximately 2.14‐fold concentrated tensile stress of 66.7 MPa. Without the stress dissipative crosslink of two disparate domains, the delicate amorphous strand (i.e., stiffness ≈ 9.9 pN Å^−1^) would preferentially be fractured and result in the brittle or ductile electrode.

Here, the degree of tyrosine crosslink was evaluated according to the tyrosine's hydrophilic/hydrophobic balanced environment.^[^ ^47^ ^]^ The tyrosine doublet (≈850 and ≈830 cm^−1^) is the characteristic Raman marker, informing the collective state of phenol hydroxy groups via hydrogen bonding (Figure 3b). The out‐of‐plane bending (2*v*_16_, 850 cm^−1^) requires more phenolic ring activity than in‐plane breathing (*v*_1_, 830 cm^−1^). Therefore, the doublet ratio (*I*_850_/*I*_830_) of 2.5 (where *I* denote Raman intensity of the Fermi resonance doublet) is the empirical parameter indicating the degree of stress dissipative tyrosine crosslink (Figure 3c).^[^ ^47^, ^48^ ^]^ In principle, the tyrosine exposed to the hydrophilic amorphous strand (*I*_850_/*I*_830_ < 2.5; blue columns in Figure 3c) constructs the hydrogen‐bonded network with the surrounding donors or acceptors (bond energy: ≈4.6–7.4 kcal mol^−1^).^[^ ^29^ ^]^ Furthermore, the crosslinked network can scatter the external stress through the anisotropic rotation and suppress the stress propagation.^[^ ^35^, ^39^, ^40^ ^]^ However, the tyrosine buried inside the hydrophobic crystallites (*I*_850_/*I*_830_ > 2.5; green columns in Figure 3c) hardly perform the interlocking and led to the slight stress dissipation. The silk fibroin electrode with the exposed tyrosine (*I*_850_/*I*_830_ < 2.5) means the sufficient crosslink between disparate crystal and amorphous strands; that is, tyrosine crosslink.^[^ ^47^ ^]^ In other words, the exposed tyrosine coincides with the hook connection with the stress dissipative spring, vice versa.

The multivalent cations (Na^+^, Ca^2+^) anchor to the polar functional groups and affect the intra‐/inter‐polymer interaction.^[^ ^49^ ^]^ Especially, the introduced divalent cation (Ca^2+^) sufficiently interacts with polar amino acids of biopolymers.^[^ ^50^ ^]^ During N‐terminal dimerization at the silk gland, the divalent cation (i.e., Ca^2+^) disturbs the electrostatic repulsion between polar functional groups (e.g., ‐COOH of Glu).^[^ ^51^ ^]^ Hence, the coordinated Ca^2+^ supports the covalent bonds within phenol rings and exposes the tyrosine out of the *β*‐sheet crystal.^[^ ^52^ ^]^ Based on the effective interactions between Ca^2+^ and phenol group of tyrosine, the tyrosine preferred to locate near the hydrophilic environment; that is, exposed state of tyrosine.

As a proof of concept, the silk fibroin electrode with high content of Ca^2+^ presented the doublet ratio in the range of ≈1.94–2.22, depicting the exposed status of tyrosine (Figure 3c). According to the fluorescence of covalently connected di‐, tri‐, and tetra‐tyrosine, the tyrosine crosslink was noticeable as the doublet ratio became below 2.5 (Figure 3d).^[^ ^53^, ^54^ ^]^ Furthermore, the silk fibroin electrode with *I*_850_/*I*_830_ < 2.5 presented the higher storage modulus and complex viscosity, informing the considerable stress dissipative network was encoded within the silk fibroin electrode (Figure 3e).

The dog‐bone‐shaped silk fibroin electrodes with various doublet ratios were subjected to the constant tensile stress of 5.0 mm min^−1^. Notably, the stress dissipation encoded silk fibroin (*I*_850_/*I*_830_ < 2.5) presented the strain versus stress curve of an elastoplastic electrode; that is, maximum stress ≥ MPa and fracture strain > 150% (blue lines in Figure 3f). Otherwise, the hardly crosslinked buried tyrosine (*I*_850_/*I*_830_ > 2.5) led to the brittle electrode; for instance, maximum stretchability < 2.0% (green lines in Figure 3f). Considering the yield strain of EPSF, the tensile recovery of EPSF is ≈31.2% which is higher than the deformation via body motions (≈30%).^[^ ^10^ ^]^ In other words, EPSF would not feature undesired plastic deformation during the daily workouts. The limit of joint movability (≈30%) is a widely employed index to evaluate the reliability of wearable electronics components.^[^ ^10^, ^14^ ^]^

When the silk fibroin electrode is elongated, the tensile load is concentrated at the amorphous domain and subsequently transferred to the crystal domain.^[^ ^18^, ^28^ ^]^ Considering the silk fibroin electrodes without tyrosine crosslink (*I*_850_/*I*_830_ > 2.5; green lines in Figure 3f), the tensile stress biased at the amorphous domain and resulted in the rapid fracture (i.e., stretchability < 2%). Therewith, the strong crystal could endure the transferred load at once (i.e., sharp maximum stress ≈ 22 MPa). On the other hand, the tyrosine crosslink could dissipate and suppress the stress concentration within the amorphous domain (*I*_850_/*I*_830_ < 2.5; blue lines in Figure 3f). Therefore, the slowly unraveling amorphous strand stretched in‐plane of elongation. Due to the encoded stress dissipation, the stretchability has surpassed the value of the theoretical amorphous strand (i.e., 150%).^[^ ^34^ ^]^ As the uncoiled amorphous chain penetrated the crystals, the crystal underwent the stick‐slip in the fulling direction.^[^ ^28^ ^]^ Thus, the crystal gradually endured the transferred load, resulting in significant elastoplasticity.

The influence of tyrosine crosslink in toughness followed the exponential curve of (toughness) = (9.74×10^2^)exp(−(doublet ratio)/0.708)−4.09 (*R* ^2^ = 0.973) (Figure 3g). Especially, the tyrosine‐crosslinked silk fibroin electrode (*I*_850_/*I*_830_ = 1.94) showed superior toughness surmounting the values of commercial fabrics (60 MJ m^−3^ of wool, 50 MJ m^−3^ of Kevlar) and elastomer (5 MJ m^−3^ of PDMS) (Figure 3g).^[^ ^18^ ^]^ Regardless of the doublet ratio, the content of *β*‐sheet crystal marginally affected the toughness. However, the distinct enhancement of toughness is originated from the stress dissipative tyrosine crosslink. For instance, the critical degree of tyrosine crosslink (i.e.*, I*_850_/*I*_830_ = 2.5) resulted in the substantial variation of toughness about 13 times. Here, the EPSF (○ = 6 wt.% and ⬡ = 1 wt.%) with the toughness of ≈ 60 MJ m^−3^ and stretchability of ≈280% was exploited to fabricate the wearable EPSF bioelectronics.

### 2.3 Harmony of Stress Dissipation Encoded Silk Fibroin Electrode with the Wearable Bioelectronics

**Figure 4:**
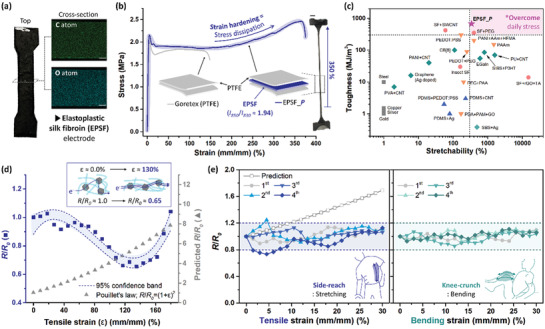
Significance of EPSF for wearable bioelectronics regarding mechanical, electrical performance. a) Photograph of dog‐bone‐shaped EPSF (ISO 527‐2, specimen type 1BA) and corresponding cross‐sectional images of atom mapping. The scale bar indicates 100 µm. b) Strain versus stress curve of the fabric bands of Goretex and EPSF_*P*. The scale bar indicates 1.0 cm. c) Mechanical properties (i.e., toughness, stretchability) of EPSF_*P* and various flexible conductors. The categorized symbols ■ (gray), ◆ (green), ▲ (blue), ▼ (orange), and ● (pink) indicated the conductors based on pristine metal, composite, elastomer, hydrogel, and silk fibroin, respectively. d) Electromechanical response of EPSF under the tension until near fracture (*ε* ≈ 180%). The symbols ■ and ▲ represent the measured and predicted resistance variations (*R*/*R*_0_), respectively. The prediction followed Pouillet's law. e) Electrical stability under the representative workouts: (left, blue) side reaching and (right, green) knee‐crunch. The cyclic experiments, in order of ●→▲→▼→◆, were performed up to the upper limit of joint movability (*ε* ≈ 30%). The dashed lines denote the reliable range of marginal *R*/*R*_0_ deviation (< 20%). The gray line (□) corresponds to the predicted values.

The electrons flow through the transportation pathway formed by the long‐range orbital overlapping of the electrical fillers.^[^ ^55^ ^]^ The homogenous electrical additive (2D carbon) through EPSF is responsible for the electrical conductivity of ≈5.33 S m^−1^, a work function of ≈4.68 eV, and an optical band gap of ≈3.81 eV (**Figure** **4**a and Figure S6, Supporting Information). The electrical conductivity was higher than the value of hard tissues (skin; *≈*0.7 S m^−1^), indicating the reduced biotic‐abiotic interface noise.^[^ ^14^ ^]^ The density of electrical filler (≈2.26 g cm^−3^) was higher than the value of silk fibroin (≈1.40 g cm^−3^).^[^ ^56^ ^]^ Hence, the gravitational sedimentation was monitored in situ as a function of filler concentration by the cross‐section image and element analysis (Figure S7, Supporting Information). The heterogeneous distribution of fillers (namely, mm‐scale sediments) considerably disturbs the mechanical characteristics.^[^ ^41^ ^]^ Therefore, the critical concentration for the filler percolation was figured out as 1.0 wt.% according to the power law of percolation theory (Figure S8, Supporting Information).^[^ ^57^ ^]^

Stress dissipation encoded EPSF is favorable in realizing the functional core‐shell fabric bands via tape coating process.^[^ ^58^ ^]^ The instant cracks of brittle core silk fibroin electrode (*I*_850_/*I*_830_ > 2.5) facilitated the failure of shell fabric due to the continuous crack propagation (Figure S9, Supporting Information). On the other hand, the core‐shell architectured EPSF fabric band (i.e., EPSF incorporated Goretex; EPSF_*P*) presented an increased performance than pristine Goretex (Figure 4b). Quantitatively, the stress dissipation from the core electrode enhanced the toughness from 265 MJ m^−3^ of Goretex to 750 MJ m^−3^ of EPSF_*P*. Hence, the tyrosine crosslink increased the strength of the core electrode up to 12.5 times and the core‐shell fabric band up to 2.89 times (Figure S10, Supporting Information). EPSF_*P* featured the strain hardening behavior after the fracture strain of shell Goretex (≈150%). Thus, the stress dissipation from core electrode to shell PTFE was responsible for the outstanding mechanics of the EPSF fabric band: toughness of 750 MJ m^−3^ and maximum stretchability of 350%. The mechanical performance of EPSF fabric band outstood the previous conductors, for instance, elastomer, hydrogel, and silk fibroin derivatives (Figure 4c). The unit components of the wearables are exposed to the deformation stress up to 450 MJ m^−3^ (Figure S11, Supporting Information). The fabrics should feature toughness over the localized stress for feasible durability. According to the stress dissipation‐based elastoplasticity, EPSF fabric band suggested the potential to endure the body motion‐induced stress. Hence, EPSF fabric band was desirable for wearable bioelectronics thanks to the harmony of stress dissipation with commercial fabrics.

Furthermore, EPSF electrodes were subjected to the axial tension to determine the electromechanical response (Figure 4d). The resistance variation (*R*/*R*_0_) of the incompressible bulk electrodes (e.g., a liquid metal) complied with the quadratic function of the tensile strain (*ε*), namely Pouillet's law: *R*/*R*_0_ = (1+*ε*)^2^.^[^ ^59^ ^]^ On the other hand, *ε* versus *R*/*R*_0_ plot of EPSF followed the cubic curve ((*R*/*R*_0_) = (5.94×10^−7^)*ε* ^3^−(1.40×10^−4^)*ε* ^2^+(0.006)*ε*+0.947; *R* ^2^ ≈ 0.91), informing the percolated filler network deformed as elongation. (Figure 4d). Furthermore, the portion of ordered conformation increased ≈193% as the strain reached ≈130% (Figure S12, Supporting Information). Hence, ≈35% reduced resistance suggested the electrical filler and *β*‐sheet crystals could be aligned along with the strain direction.^[^ ^60^ ^]^

Since the elasticity of EPSF was determined as ≈31.2%, the motional stabilities were investigated by simulating the physical deformations during the representative workouts. The wearable bioelectronics frequently encounters stretching (side‐reach) and bending (knee‐crunch) deformations during the workout routine (Figure 4e). The maximum strains for both motions were 30%, according to the normal range of motion.^[^ ^9^, ^10^ ^]^ EPSF presented stable electrical properties when two types of stress were applied repetitively. Quantitatively, *R*/*R*_0_ was in the range of 0.8–1.2, and the linear deviation was suppressed ≈85.2% compared to the theoretical bulk electrode (Figure S13, Supporting Information).^[^ ^29^ ^]^ Thus, EPSF could be the potential core electrode of core‐shell fabric to implement wearable bioelectronics.

### 2.4 Model Device Studies about Triboelectric Behaviors of EPSF Bioelectronics

The prototype of EPSF bioelectronics was selected as the triboelectric wearables utilizing electricity during workouts. Specifically, the stress dissipation encoded EPSFs were harnessed as the core electrode of energy harvesting fabric bands comprising the wearables. Triboelectrification produces a continual electrical output due to the spontaneous surface frictions of fabric bands originating from body motions. Prior studies have proposed various triboelectric fiber or fabric band‐based clothes and textiles by exploiting the material library in the triboelectric series.^[^ ^60^, ^61^ ^]^ However, the sophisticated mechanism of electricity generation has been hardly established owing to the cumbersome woven structure.^[^ ^62^ ^]^ EPSF fabric band enabled the realization of the realistic woven structure without creeping or bumping (Figure S11, Supporting Information). Hence, the detailed electricity generation mechanism was discussed, contemplating the woven system.

The suitability of EPSF for triboelectrification was evaluated according to the parametric studies of single electrode mode. Figure S14, Supporting Information, elaborated diverse conformations' electrical outputs—peak open‐circuit voltage (*V*_OC_) and peak closed‐circuit current (*I*_CC_). Regarding the first configuration, each commercial aluminum (Al) and EPSF electrode, which is packaged by PTFE and contacted with nylon, has generated a maximum *V*_OC_ of 43.2 and 51.2 V, and maximum *I*_CC_ of 2.8 and 3.3 µA (Figure S14a,b, Supporting Information). In the opposite event that each Al and EPSF is incorporated with nylon and undergoes vertical contact with PTFE, Al has produced a maximum peak *V*_OC_ of 30.4 V and peak *I*_CC_ of 3.2 µA. Still, EPSF has generated a maximum peak *V*_OC_ of 36 V and peak *I*_CC_ of 3.8 µA (Figure S14c,d, Supporting Information). As a result, the comparable electrical performances (deviation < 20%) are featured irrespective of the polarity of surface charge (namely, polar directions of peaks), suggesting that EPSF is capable of behaving as an electrode similar to the aluminum.

**Figure 5:**
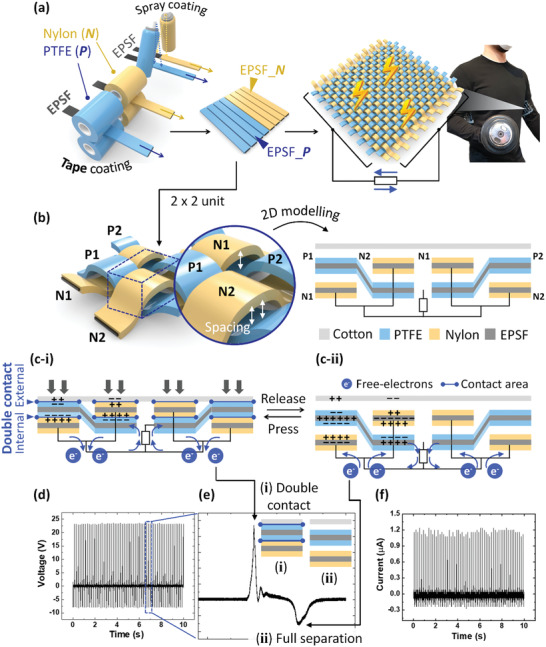
Fabrication and characterization of the EPSF bioelectronics. a) The practical procedure (i.e., tape or spray coating) has been applied to produce the core‐shell architectured EPSF fabric bands. EPSF incorporated Goretex (EPSF_*P*), and EPSF incorporated nylon (EPSF_*N*) were created. EPSF_*P* (warp) and EPSF_*N* (weft) are weaved as the piece of sportswear. b) 3D and 2D modeling of a 2 × 2 unit featuring the woven structure. c) The working mechanism of a 2 × 2 unit has contemplated the double contact and separation within the realistic woven system. The charges on the right sides of (c‐i) and (c‐ii) were skipped; however, the right side corresponds to the mirror effect of the left side. Triboelectric performance of 2 × 2 units. d,e) Peak open‐circuit voltage (*V*_OC_) output plot and corresponding magnification of *V*_OC_. f) Peak closed‐circuit current (*I*_CC_) outputs.

**Figure** **5**a illustrates the fabrication process from EPSF to wearable bioelectronics. Core EPSFs (diameter ≈ 12 cm) were sliced into the designed shape of the fabric band. Processed EPSFs were packaged with the commercial fabrics via the practical tape coating method. Specifically, 1.0 cm × 6.0 cm × 500 µm (width × length × thickness) EPSFs were incorporated with Goretex (*P*) or nylon (*N*) tapes. Goretex (PTFE) and nylon are tribo‐polarity elements. EPSF incorporated Goretex (EPSF_*P*), and EPSF incorporated nylon (EPSF_*N*) correspond to the warp and weft fabric band, respectively. EPSF_*P* and _*N* were plain‐woven to form a piece of cloth; after that, it was introduced into commercial sportswear.

The clothes naturally possess a woven structure. Figure 5b presents the 3D and 2D modeling of a 2 × 2 unit of the woven system. 2 × 2 unit means when two warps and weft are crossed to present the plain weave geometry (Figure S15a, Supporting Information). The characteristic of woven structure was considered; that is, the vertical spacing between EPSF_*P* (P1 or P2) and EPSF_*N* (N1 or N2). According to the vertical spacing, the 2 × 2 unit simultaneously underwent double contact, i.e., external and internal contacts (Figure 5c). At external contact with the cotton, EPSF_*P* and EPSF_*N* were negatively and positively charged by triboelectrification. Hence, the negative and positive charges were generated on EPSF_*P* and EPSF_*N* surfaces due to internal contact within the fabric bands. During the press (double contact, Figure 5c‐i) and release (separation, Figure 5c‐ii) cycle, the flow of free‐electrons occurred via electrostatic induction effect and generated a maximum *V*_OC_ of 23.6 V (Figure 5d). In Figure 5e, the representative voltage waveform of a single‐cycle involves two distinct peaks of double contact and separation. Additionally, the 2 × 2 unit produced the maximum peak *I*_CC_ of 1.24 µA (Figure 5f and Figure S14b, Supporting Information). The reflection of realistic woven structure (i.e., double contact) results in a noticeable difference in triboelectric outputs. Figure S16, Supporting Information, presented the simplified working mechanism on single contact that hardly considered woven structure and corresponding output voltage plot. The maximum *V*_OC_ of the single contact model device is ≈6.5 times lower than the value of the double contact mechanism.

The material selection is performed according to the triboelectric material library (i.e., triboelectric series) (Figure S17, Supporting Information). Hence, the most negative and positive fabrics (i.e., PTFE and nylon) were exploited to package EPSFs (Figure S17a, Supporting Information). Owing to the significant electron affinity difference, the packaged EPSFs can be easily charged via internal contacts (Figure S17a‐iii, Supporting Information). Furthermore, cotton was selected as a counterpart material for external contact (Figure S17a‐i, ii, Supporting Information) due to its neutral position in the triboelectric series. Thanks to the compensation by the external contact, the selection of counterpart materials are relatively diverse (Figure S17b,c, Supporting Information). Meanwhile, the similar electron affinity of packaging materials should be restrained; that is, it significantly influences the electrostatic induction profile (Figure S17d, Supporting Information). Furthermore, the irregular contact‐separation frequency originating from the various body motions positively affects the electrical outputs. For example, the maximum *I*_CC_ output was enhanced from 1.24 to 2.16 µA, but the maximum *V*_OC_ of 23 V was consistent as the frequency increased from 3 to 6 Hz (Figure S18, Supporting Information), following the previous studies.^[^ ^61^ ^]^

EPSF fabric bands were woven into the larger surface area (i.e., 5 × 5 fabric band device in Figure S19a, Supporting Information) and introduced in the near‐armpit areas of commercial cotton sportswear (Figure 5a). During various workouts, the triboelectric outputs of 5 × 5 fabric band devices can be exploited as the power source of wearable electronics: electrostimulation and motion sensor. The representative workouts generated the maximum *V*_OC_ of 11.2 V during the vertical motion (side lateral raise in Figure S19b, Supporting Information) and 8.6 V during the sliding motion (running in Figure S19c, Supporting Information). The state‐of‐the‐art studies found that external electrical signals (ca. 10 V) across the mechanoreceptors restore the cutaneous haptic sensation.^[^ ^63^ ^]^ Furthermore, sufficient electrical energy opened the voltage‐gated Ca^2+^ channels in the plasma membrane and stimulated the individual cell activity.^[^ ^64^ ^]^

### 2.5 Futuristic Functions of EPSF Bioelectronics: Wearable Personal Trainer

**Figure 6:**
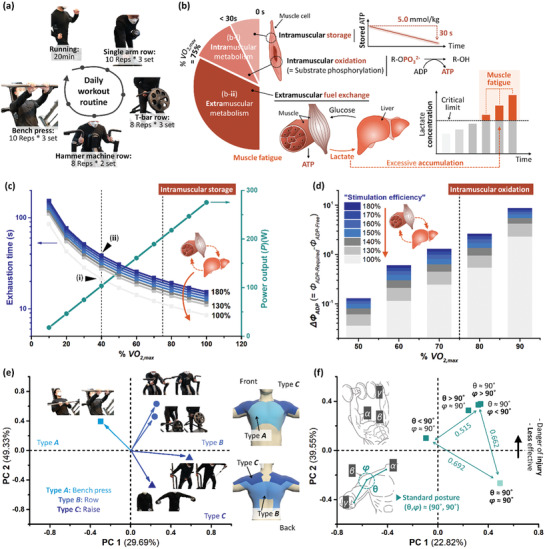
Demonstrative application of the EPSF bioelectronics in daily workouts. a) The professional athlete wearing EPSF bioelectronics worked out following the personalized routine: warming up (running), three types of weightlifting for the dorsi growth, and the floorwork (bench press). The routine was performed following the quarantine guidelines. b) Skeletal muscle metabolism for sustained biofuel supply, that is, ATP, during aerobic workouts. The cellular event could manage the whole body efficiency. (b‐i) The intramuscular metabolism consumed the stored ATP within the first 30 s. Therewith, the activated phosphorylation metabolism (i.e., intramuscular phosphorylation) replenished the deficient ATP. Adenosine diphosphate (ADP) is the representative substrate for intramuscular phosphorylation for submaximal workouts (% *VO*_2,max_ < 75%). (b‐ii) According to the increased intensity of muscular contraction, the fuel exchanges from the extramuscular organ (e.g., liver glycogen) become vital. The byproduct of extramuscular metabolism was lactate accumulation, accompanying the widespread muscle fatigue (e.g., myalgia). c,d) Mathematical and theoretical biology approaches to compute the delayed onset of muscular fatigue. (c) The linear correlation of workout intensity (% *VO*_2,max_) and power output (*P*) is plotted with a green circle (●). The delayed exhaustion times (■) are calculated as a function of workout intensity and stimulation efficiency from 100% to 180%. (d) The difference between free and required ADP concentration (Δ*Φ*_ADP_) was derived to elucidate the extramuscular metabolism rate. The higher Δ*Φ*_ADP_ indicates the enhanced fuel exchange rate, revealing accelerated fatigue. e,f) Sensory functions of EPSF bioelectronics: (e) Inform the obvious muscle by performing resistance exercise and (f) warn the risky postures to inhibit the severe muscular injuries for the amateurs. The green values in Figure 6f correspond to the rectilinear distances between the farthest data points.

In **Figure** **6**, the mathematical and theoretical biology and statistical investigation elicit the potential leverages when performing the active mobility with EPSF bioelectronics (i.e., smart wearables in Figure 5). The widespread muscle fatigue due to muscular (e.g., chronic/temporary myalgia and muscle‐burning sensation) overload impedes the ideal workout performance and suppresses the passion for repetitive fitness. Besides, improper weightlifting can cause severe musculoskeletal injuries such as fractures, dislocations, and herniated disks. Therefore, non‐professional athletes favor hiring PTs. Specifically, PT coaches the amateur toward a personally optimal workout routine preventing muscle overload and standard postures to get out of the critical incidence.^[^ ^25^ ^]^ However, the financial matter has been issued since the average cost of PT was $40 to $70 per hour session (e.g., $60 in Los Angeles, 2021). Considering the active mobility of daily workout routine (Figure 6a), the conceptual application of the EPSF bioelectronics, so‐called wearable personal trainer (WPT), was evaluated. The stress dissipative warp (EPSF_*P*) and weft (EPSF_*N*) are capable of enduring the curvilinear fractures; therefore, no significant deformation has occurred after the dynamic motions. Moreover, EPSF_*P* and _*N* present no cytotoxicity to the fibroblast (i.e., human dermal fibroblast (HDF)) and myoblast (i.e., C2C12) (Figure S20, Supporting Information).

The continual production of adenosine triphosphate (ATP) through the cellular metabolism is the principal homeostasis to compensate for the energy expenditure by the muscle contractile activities (Figure S21, Supporting Information). In detail, the muscular events (consumption of the stored ATP → phosphorylation → fuel exchange) mutually compensated the ATP utilization for the workouts (Figure 6b). The first two represent the intramuscular metabolism (Figure 6b‐i), and the last corresponds to the extramuscular metabolism (Figure 6b‐ii).^[^ ^65^ ^]^ The expeditious fuel exchange with the liver (or adipose tissue) leads to hyperlactatemia as the muscular contraction intensity increases.^[^ ^65^ ^]^ The lactate accumulation above the homeostatic level caused the lack of oxygen‐rich blood and accompanied the widespread muscle pain (namely, myalgia^[^ ^66^ ^]^) that originated from acidosis. Therefore, to relieve muscle fatigue, ATP supplied from the intramuscular metabolism should be formidable, ideally, irrespective of the exercise intensity. Here, the percentage versus the maximal mass rate of oxygen uptake (% *VO*_2,max_) corresponds to the desired ATP utilization rate and exercise intensity (Figure S21, Supporting Information).^[^ ^65^ ^]^

The external electrical signals can enhance cellular activity, that is, electrostimulation.^[^ ^67^ ^]^ For instance, when the electric field induced Ca^2+^ uptake through the plasma membrane, the phosphorylase kinase transformed from a less active “*b*” form to a more active “*a*” state.^[^ ^65^ ^]^ Therefore, electrostimulation can support the extension of intramuscular metabolism. In Figure S22a,b, Supporting Information, the triboelectricity from WPT stimulated approximately 130% to 180% of the fibroblast and myoblasts in vitro. As a proof of concept, the synthesis of growth factors and vital proteins was upregulated. In detail, the level of basic fibroblast growth factor (bFGF) was enhanced by approximately 140% (Figure S22c, Supporting Information). The expeditious release of insulin‐like growth factor 1 (IGF‐1) was manifested up to 180% (Figure S22d, Supporting Information). Furthermore, protein secretion (i.e., type I collagen) became abundant at least 150% (Figure S22e,f, Supporting Information). Therefore, the electrical signals stimulated the fibroblast and myoblast activity, that is, electrostimulation (Figure S22f, Supporting Information). Here, the model growth factors and protein are crucial in aerobic muscular metabolism. For instance, bFGF triggers the mitogen‐activated protein kinase signaling pathway. The type I collagen influences the junction of connective tissue, that is, the recovery of muscular tubes from muscle fatigue. IGF‐1 facilitates the differentiation of the myoblast toward the tubular formation.

The intramuscular ATP storage is ≈5.0 mmol kg^−1^ and would exhaust within 21.2 s during submaximal exercise (Figure 6c‐i and Figure S21, Supporting Information). The depleted ATP would be compensated by the extramuscular metabolism, meaning muscle fatigue. Regarding ATP storage to be proportional to cellular activity, the exhaustion with WPT occurs after 38.3 s to conduct the identical performance (power output ≈ 103.6 W) (Figure 6c‐ii and Figure S21, Supporting Information).

Another intramuscular metabolism, aerobic substrate‐level phosphorylation (i.e., glycolysis), demands the substrate (i.e., adenosine diphosphate (ADP)) to synthesize ATP for contracting the skeletal muscle. The free ADP concentration (*Φ*_ADP‐Free_) kept ≈0.251 mmol kg^−1^ owing to the homeostasis.^[^ ^68^ ^]^ The required ADP concentration to perform the desired exercise intensity (*Φ*_ADP‐Required_) was estimated from the second‐order Michaels–Menten kinetics (Equation (3)). Figure S23, Supporting Information, elucidates the details of Equation (3).

(3) $$\Phi_{\mathrm{ADP}-\mathrm{Required}}^{2}=\frac{\left(K_{s1}\right)\left[\left(P\right)\left(K_{s4}\right)+\left(W\right)\left(VO_{2}\right)\right]}{\left(W\right)\left(VO_{2,\max}\right)-\left(P\right)\left(K_{s4}\right)-\left(W\right)\left(VO_{2}\right)}$$

where *K*_S1_ represents the 50% activity constant of phosphorylation (0.0631 mmol^2^ kg^−2^). Besides, *P* and *W* denote the power output and the body weight, respectively (Figure S21, Supporting Information); *K*_s4_ denotes the oxygen‐workload constant. *Φ*_ADP‐Required_ is dependent on *K*_s4_ because the respiratory exchange ratio collected at the mouth represented the metabolic activity at mitochondrion (e.g., spirometry device).

The difference between free and required ADP concentration (Δ*Φ*_ADP_ = *Φ*_ADP‐Required_−*Φ*_ADP‐Free_) addresses whether the fuel exchange with the extramuscular substrates should be triggered to fulfill the ATP demand of a specific workout (Figure 6d). For example, Δ*Φ*_ADP_ < 0 indicates that the homeostasis is sufficient in the moderate‐intensity (% *VO*_2,max_ ≤ 40%). However, the extramuscular metabolism should be activated to maintain the ATP resynthesis rates to perform the submaximal/maximal muscle contractile event (Δ*Φ*_ADP_ > 0, Figure S23, Supporting Information). In other words, the possibility of muscle fatigue (i.e., the byproduct of extramuscular metabolism) corresponds to the column area in Figure 6d. From the mathematical and theoretical biology investigations, the widespread muscle fatigue due to hyperlactatemia can be delayed significantly when the workout is supported by active electrical energy from WPT.

The proposed EPSF bioelectronics (WPT) can transmit the sensory information adequate for non‐professional athletes. The principal component analysis (PCA) is adopted to reduce the dimensionality of waveforms and statistically evaluate the triboelectric outputs during the workout (Figure 6e,f). The rectilinear distance within the scatters addresses the statistical deviation in the original data set; that is, 0.692 corresponded to a significant difference than 0.515 in Figure 6f.^[^ ^69^ ^]^ In Figure 6e, the representative workouts were categorized in terms of the muscle growth spots. Specifically, type *A*, *B*, and *C* is associated with the pectorals, latissimus dorsi, and deltoid, respectively. The body would behave analogously if the objective muscle growth regions were in the comparable section. The similar behavior led to statistically comparable triboelectric outputs. According to the realistic problems, the appropriate or preferred workout methodologies are personalized. Therefore, WPT can inform amateurs about the ideal stimulation region of performing resistance exercises even though they exploited the various machines (● in Figure 6e) or favored the free weight (▲ Figure 6e). Furthermore, the durable WPT coached the standard posture, enabling to diminish the muscular injuries. The incline dumbbell press was performed in multiple angles (*θ*, *φ*), the non‐dangerous range for the technical subject, as shown in Figure 6f. The standard posture of the model workout was defined as (*θ*, *φ*) ≈ (90°, 90°). Notably, WPT classified the appropriate or risky postures (Figure 6f). Therefore, the athlete may potentially recognize the accuracy of posture and get out of the injury.

## 3 Conclusion

Here, we have encoded the functionality of stress dissipation within the silk fibroin electrode by realizing the constructive role of phenolic amino acids. On the other hand, flexible polymeric conductors have been suggested with the help of external crosslinkers. Prior studies introduced the strong hard segments into a flexible matrix via copolymerization, blending, or double network for mechanical performance.^[^ ^16^, ^17^ ^]^ However, the physical (e.g., van der Waals interaction ≈ 1 kT) or chemical (e.g., covalent bond ≈ 80 kT) crosslinks were requisite to circumvent the phase separations originated from the interfacial mismatch among elements. The excessive crosslink guides the brittleness of polymer electrodes, meaning the trade‐off relationship between toughness and stretchability.

We have focused on the intrinsic functionality of canonical amino acid to bottom‐up design the stress dissipation encoded silk fibroin electrode. In detail, the stress propagation within protein secondary structures was manipulated as the function of tyrosine crosslink. The degree of tyrosine crosslink was visualized via Raman scatterings. Significantly, the specific Raman marker (i.e.*, I*_850_/*I*_830_ < 2.5) enabled us to distinguish the degree of dissipation. Hence, the sufficient tyrosine crosslink (*I*_850_/*I*_830_ = 1.94) enhanced the toughness about ≈12.5 times, indicating the considerable encoding of stress dissipation within the silk fibroin electrode.

Regarding the core‐shell fabric bands, the substantial stress dissipation of the core EPSF electrode led to the shear hardening response. Thus, the superior performance was realized: toughness ≈ 750 MJ m^−3^ and stretchability ≈ 350%. Considering the daily motions with wearable bioelectronics, the localized intersections of warp and weft underwent substantial stress up to 450 MJ m^−3^. Hence, EPSF fabric bands were anticipated to overcome the motion‐induced stress, informing the feasible weavability of EPSF fabric bands.

The triboelectric wearables were proposed as the prototype of EPSF bioelectronics to satisfy the demands of athletic populations. The detailed mechanism of energy harvesting was figured out considering the weaved structure of the prototype. According to the innovative studies about EPSF bioelectronics, the users can perform active workouts with suppressed muscular fatigue and injuries. The proposed concept might open the door to futuristic bioelectronics regardless of the lifestyle (e.g., athleisure or cozy daily looks).

In this study, the uncharted strategy of intrinsic crosslink through biopolymer matrix was established considering the multivalent amino acid. The distribution of secondary structure is highly distinct within the protein types. Hence, the *β*‐sheet crystallite technology of silk fibroin is hardly applicable to the hair‐derived keratin mainly comprised of *α*‐helix coiled‐coil architecture. Remarkably, the potential applications and significance of amino acid crosslinker technology are inexhaustible. The library of amino acid crosslinkers could be broadened to diverse canonical building blocks of subject protein involving the polar side chains, for example, serine, threonine, asparagine, glutamine.

## 4 Experimental Section

Experimental details are provided in the Supporting Information.

## Conflict of Interest

The authors declare no conflict of interest.

## Supporting information

Supporting Information

Supplemental Video 1

Supplemental Video 2

## Data Availability

The data that support the findings of this study are available from the corresponding author upon reasonable request.
