# Supplementary material for: Stress Dissipation Encoded Silk Fibroin Electrode for the Athlete‐Beneficial Silk Bioelectronics
Source: Adv Sci (Weinh). 2022 Jan 9;9(8):2105420. doi: 10.1002/advs.202105420 (PMC8922117; doi:10.1002/advs.202105420)
Supplement: Supplementary file 1 — Supporting Information [file ADVS-9-2105420-s001.pdf]

ADVANCED SCIENCE
Open Access

Supporting Information

for *Adv. Sci.*, DOI 10.1002/advs.202105420

Stress Dissipation Encoded Silk Fibroin Electrode for the Athlete-Beneficial Silk Bioelectronics

Woojin Choi, Deokjae Heo, Taeho Kim, Sungwon Jung, Moonhyun Choi, Jiwoong Heo, Jae-Sung Kwon, Byeong-Su Kim, Wonhwa Lee, Won-Gun Koh, Jeong Ho Cho, Sangmin Lee and Jinkee Hong**

ADVANCED SCIENCE
Open Access

Supporting Information

for *Adv. Sci.,* DOI: 10.1002/advs.202105420

Stress Dissipation Encoded Silk Fibroin Electrode for the Athlete-beneficial Silk Bioelectronics

Woojin Choi, *Deokjae Heo*, *Taeho Kim*, *Sungwon Jung*, *Moonhyun Choi*, *Jiwoong Heo*, *Jae-Sung Kwon*, *Byeong-Su Kim*, *Wonhwa Lee*, *Won-Gun Koh*, *Jeong Ho Cho*, *Sangmin Lee**, and *Jinkee Hong**

Supporting Information

Stress Dissipation Encoded Silk Fibroin Electrode for the Athlete-beneficial Silk Bioelectronics

Woojin Choi†, *Deokjae Heo*†, *Taeho Kim*, *Sungwon Jung*, *Moonhyun Choi*, *Jiwoong Heo*, *Jae-Sung Kwon*, *Byeong-Su Kim*, *Wonhwa Lee*, *Won-Gun Koh*, *Jeong Ho Cho*, *Sangmin Lee**, and *Jinkee Hong**

W. Choi, T. Kim, S. Jung, M. Choi, J. Heo, Prof.. W. –G. K, Prof. J. H. Cho, and Prof. J. Hong
Department of Chemical and Biomolecular Engineering, College of Engineering, Yonsei University, Seoul 03722, Republic of Korea
E-mail: jinkee.hong@yonsei.ac.kr

D. Heo, and Prof. S. Lee
School of Mechanical Engineering, Chung-ang University, 84, Heukseok-ro, Dongjak-gu, Seoul 03722, Republic of Korea
E-mail: slee98@cau.ac.kr

Prof. J. –S. Kwon
Department and Research Institute of Dental Biomaterials and Bioengineering and BK21 FOUR Project, Yonsei University College of Dentistry, Seoul 03722, Republic of Korea

Prof. B. –S. Kim
Department of Chemistry, Yonsei University, Seoul 03722, Republic of Korea

Dr. W. Lee
Department of Chemistry, Sungkyunkwan University, Suwon 16419, Republic of Korea

† Both authors contributed equally to this work.

*Correspondence authors: Prof. Jinkee Hong (E-mail: jinkee.hong@yonsei.ac.kr) and Prof. Sangmin Lee (E-mail: slee98@cau.ac.kr)

Supplementary Information Content

► Experimental Section / Methods

► Supporting Figures

► References

Experimental Section / Methods

Synthesis and characterization of the electrical filler. The hydrothermal reaction was performed to synthesize the β-chitin-derived-carbon. Inside the commercial hydrothermal reactor, 1.5 g β-chitin (Sigma-Aldrich, St. Louis. MO, USA) was dispersed in the 20 mL deionized water. The hydrothermal reaction proceeded at 200 °C for 6 h. The black and viscous residue was washed by 9,000 rpm centrifugation (VARJISPIN 15, CRYSTE, Korea). Thereafter, the β-chitin-derived-carbon powder was gathered by overnight lyophilization. The operation condition of the lyophilizer (FreeZone 2.5 L Benchtop Freeze Dry System #7670540, LABCONCO, USA) was -50 °C and 0.06 bar. The β-chitin-derived-carbon powder was subjected to the physical reduction at 850 °C for 2 h in the argon atmosphere. In the tube furnace, the heating rate was 10 °C min^{-1} to reach the target temperature. The obtained black powder was named as reduced β-chitin-derived-carbon (rCDC). The gram yield of rCDC was ~25% compared to the initial β-chitin amount. In order to analyze the D/G ratio, Raman spectroscopy (XploRA PLUS, HORIBA, France) was performed. For Raman studies, 100× microscope objective (numerical aperture = 0.25) and 532 nm laser were selected. Besides, 1,800 grating spectrometer was exploited with a confocal of 100 μm. The laser intensity at the sample point was ~75 mW; the diameter of the laser spot was 2.6 μm. The sharp peak of the crystalline silicon at 520 cm^{-1} was the standard peak for the calibration before the measurements. The laser was exposed for 30 s; the measurement was repeated five times to obtain reliable results. The high-resolution X-ray diffractometer (XRD; SmartLab, Rigaku,

Japan) was conducted for the crystallographic investigation of rCDC. The high-performance X-ray generator (9 kW) was embarked. rCDC was uniformly transferred on 1 × 1 cm^2 silicon wafer. The standard alignment was based on a 5.0 mm length limiting slit for accurate measurement. The step for each measurement was 0.02°, and the scan rate was 3° min^{-1}. Furthermore, X-ray photoelectron spectroscopy (XPS; K-ALPHA, Thermo Fisher Scientific, USA) was conducted to elicit the atomic characteristics of rCDC.

Fabrication of silk fibroin electrode. Silk cocoons were purchased from the local market and degummed following the previous protocol.[1] Briefly, the silk cocoons were cut into coin size. The sliced silk cocoons were poured into the boiling 0.02 M Na_2CO_3 bath (at least 90 °C) and degummed for 1 h. The degummed silk fibroin was thoroughly rinsed in deionized water and dried overnight under ambient condition. The formic acid with $CaCl_2$ (3 or 6 wt%) (Sigma-Aldrich, St. Louis. MO, USA) and rCDC (0 or 1 wt%) was used as the dissolving reagent of the degummed silk fibroin. We rationally determined 1 wt% of rCDC regarding the cross-section images of field emission scanning electron microscope (SEM; IT-500HR, JEOL, Japan) and energy dispersive spectrometry (EDS; 7610f-plus, JEOL, Japan). The homogenous dissolving reagent was obtained by 1 h sonication. The dissolving reagent was black in color due to the well-distributed rCDC. After that, the degummed silk fibroin (10 wt%) was fully dissolved by the prepared dissolving reagent at 60 °C for 1 h. The dissolved silk fibroins were cast on the polystyrene mold in the appropriate size (with 3.5, 5.0, and 10.5 cm diameter) and overnight dried under the ambient condition (~25 °C and relative humidity of ~ 65%). The thermal treatment (60 °C for 2 h) of the dried silk fibroin films facilitated the detachment from the polystyrene mold.[2] The brittle and elastoplastic silk fibroin electrode (BSF and EPSF) referred to the samples prepared with 3 and 6 wt% $CaCl_2$, respectively. The fabricated silk fibroin electrodes (BSF, EPSF) were vacuum packaged and stored inside the desiccator (relative humidity ≈ 20%; Dryactive, Kastech, Korea) until the characterizations or further procedures.

Investigation of the electromechanical response under the mechanical load. To concentrate the tensile stress, EPSF was shaped into the dog bone gauge regarding ISO 527-2, 1BA specimen type (Figure 4a). A universal testing machine (UTM; Model 3366, Instron, USA) was used to apply the uniform tensile and bending load to EPSF. For the tension experiment, the gripped region was 50 mm^2 and the tensile rate was 5 mm min^{-1}. The tension was kept to the maximum strain in Figure 4d. UTM released the specimen when the stain reached 30% and re-stretched the specimen in Figure 4e. The cyclic test was repeated four times. Besides, the bending test was performed on the beam gauge in 60 × 10 mm^2 (width × length). The bending rate (*i.e.,* crosshead speed) was 5 mm min^{-1}. The cyclic test was conducted, as mentioned above. The relative humidity was kept at 50% during the mechanical deformation. In situ variation of resistance was measured by the multimeter (Fluke 179, Fluke, USA).[2] Pre-stretched EPSF ($\varepsilon \approx 130\%$) was fixed and vacuum-stored to investigate the crystal properties by Raman spectroscopy.

Multilateral characterizations of silk fibroin electrode. The electronic level of silk fibroin electrode was elicited using kelvin probe force microscopy (KPFM; NX-10, Park systems) and UV-visible spectroscopy (UV-Vis; Evolution 300, Thermo Scientific, USA). KPFM was conducted to measure the work function. The non-contact tip was coated with the platinum with a tip size of 27.5 × 255 × 15 μm^3 (width × length × height). The resonance frequency, spring constant, and sensitivity were 75 kHz, 2.8 N m^{-1}, and 33.3 V μm^{-1}, respectively. The driving voltage was 2.0 V. 10 × 10 μm^2 measurement area was divided into 512 pixels and scanned at a 1.00 Hz rate. Prior to the KPFM measurement of silk fibroin electrodes, the work function of highly ordered pyrolytic graphite was estimated as the standard. The optical band gap was calculated from UV-Vis spectra over the range of 200 to 900 nm. The temperature was kept at 25 °C by means of the thermoset accessory. The bandwidth, data interval, and scan rate were 2.0 nm, 1.0 nm, and 200 nm min^{-1}, respectively. Xenon lamp was the light source, and the standard baseline was measured by the empty cell (1 cm length). Fourier

transform infrared spectroscopy (FT-IR; FT/IR-4700, JASCO, Japan) was performed to monitor the vibrations of amino acids. Additionally, Raman spectroscopy and XRD were conducted to monitor the inherent characteristics of 1 × 1 cm^2 silk fibroin electrode (*e.g.,* crystal properties and tyrosine environment). The experimental protocol was the identical as mentioned above. 1400 to 1500 cm^{-1}, 1640 to 1720 cm^{-1}, 800 to 900 cm^{-1} spectra individually are related to amide III, amide I, and tyrosine doublet, respectively. XRD pattern in the range of 3 to 40° informed the (110) direction of the β-sheet crystal. Furthermore, the fluorescence spectrometer (FP-8300, Jasco, Japan) and rheometer (MCR 302, Anton Paar, Austria) were conducted for the in-depth characterizations. The silk fibroin electrodes (2 cm diameter) were subjected to excitation at 315 nm. The emission was recorded at 350 to 500 nm in 2 nm increments. The scan rate was 500 nm min^{-1}, and the slits were set to yield sufficient fluorescence resolution (*ca.* 2.5 nm). The frequency-dependent storage modulus of 1 × 1 cm^2 silk fibroin electrode was measured under the frequency sweep mode (set as 1.0% strain amplitude) and the constant temperature of 25°C. The gap was 500 μm, corresponding to the thickness. Finite element simulation was conducted with a commercial software ABAQUS (ABAQUS Inc. Student edition). The unit silk fibroin was considered as the elastic beam. The detailed parameters utilized in the simulation are summarized in Table S1. Engineering strain *vs.* stress curves was recorded during the tension using the UTM. The detailed experimental protocol (*e.g.,* tensile rate) was accorded with the above.

Fabrication of EPSF-incorporated triboelectric fabric band. EPSFs were cut into ten pieces of 1 × 6 cm^2 each. Every piece was fully packaged using commercial PTFE (thickness of 0.08 mm, Chukoh Chemical Industries, Korea) and nylon (thickness of 0.05 mm, KAWAGUCHI, Korea) tapes. PTFE-packaged EPSF (EPSF_*P*) and Nylon-packaged EPSF (EPSF_*N*)—corresponding to warp and weft fabric band—were woven into a plain weave pattern. The final sample of the 5 × 5 fabric band has a natural woven structure.

Characterization of triboelectricity. Vertical excitation input was applied to a 5 × 5 fabric band device using a vibration tester (ET-126B-4, Labworks, USA), a function generator (AFG3021C, Tektronix, USA), and an amplifier (pa-151, Labworks, USA). The vertical vibration amplitude and frequency were set at 3 mm and 6 Hz, respectively. The voltage output and current output were measured using a mixed domain oscilloscope (MDO 3014, Tektronix Co, USA) and a low-noise current preamplifier (MODEL SR570, Stanford Research Systems, USA), respectively. The voltage probe 1 and 2 were connected to the warps (EPSF_*P*) and wefts (EPSF_*N*). The voltage output was obtained from the electrical potential difference between probes 1 and 2. The current output was measured exploiting one current probe, directly connected with the oscilloscope. Principal component analysis (PCA) was conducted as the statistical approach to evaluate the obtained waveform.

In vitro cell experiments. Here, the fibroblast (*e.g.,* human dermal fibroblast (HDF)) and myoblast (C2C12) were the model cell lines that are essential in the aerobic resistance exercise. Prior to the various assays, the model cells were fully sub-cultured and stabilized by incubation at 37 °C in a humidified incubator with 5% CO_2 in Dulbecco's modified Eagle medium supplemented with 10% fetal bovine serum (Gibco Life Technologies, USA). To verify the no cytotoxicity of EPSF_*P* and EPSF_*N*, prewashed fabric bands of 1 × 1 cm^2 were thoroughly sterilized by the overnight UV exposure and transferred 24-well culture plate. The cells (HDF and C2C12) with a density of 5.0×10^4 cell mL^{-1} were seeded in each well with a pre-sterilized fabric band. After 24 h incubation, a cell counting kit assay (CCK assay: D-Plus CCK cell viability assay kit, Dongin LS, Korea) was performed following the manufacture's protocol. Specifically, the wells were treated with 10% (v/v) CCK assay solution and incubated for 2 h. The relative viability was monitored by tracking the optical density at 450 nm. The lab-made multi-well culture plate, assembly of indium tin oxide (10 × 10 cm^2), and removable chambers (three well chambers, ibidi) were exploited for *in vitro* electrostimulation experiments. Before the cell culture, the lab-made culture plates were

sterilized deliberately with 70% (v/v) ethanol and UV exposure. The power source of electrostimulation was triboelectricity of 5 × 5 fabric band device or equivalent triboelectricity (*i.e.,* $V_{OC} \approx 20$ V with 6 Hz frequency). Both sides of the culture plate were individually connected with the warp (EPSF_*P*) and weft (EPSF_*N*) of the 5 × 5 fabric band device inserted in the vertical vibration tester. Moreover, HDF and C2C12 (in 4.0×10^4 cell mL^{-1}) were incubated overnight for sufficient adherence. After that, 2, 4, and 6 h of electrostimulations (n=3) were performed according to triboelectricity of 5 × 5 fabric band. 10% (v/v) CCK assay solution was treated and incubated for 2 h simultaneously with electrostimulation to analyze the cellular dehydrogenase activity. The optical density was measured as described above. The enzyme-linked immunosorbent assay (ELISA) was performed to quantify the amount of secreted protein and growth factor considering the origin of model cells. The cell culture supernatant of HDF (1 mL) directly after the electrostimulation was subject to the type I collagen ELISA (Human Pro-Collagen I alpha, R&D systems, USA and Mouse Pro-Collagen I alpha, abcam, USA). Furthermore, the lysis procedure was followed to quantify the amount of basic fibroblast growth factor (bFGF) from HDF (Human FGF ELISA Kit, Thermo Fisher, Scientific, USA) and the insulin-like growth factor 1 (IGF-1) from C2C12 (Mouse IGF-1 ELISA Kit, Thermo Fisher, USA). In detail, the electrostimulated cells were gathered from 10% (v/v) trypsin-EDTA solution and exposed to the pH 7.4 icy lysis buffer for 30 min. The component of NP-40 lysis buffer was 150 mM NaCl, 1% (v/v) Triton X 100, and 50 mM Tris-Cl. The cleaved plasma membranes were pellet down by 13,000 rpm centrifugation for 10 min. Processed supernatant was subject to bFGF and IGF-1 ELISA. All ELISAs were proceeded according to the manufacturer's protocol. The supernatants from culture media or lysis buffer were diluted 1/20 (type I collagen from HDF; $R^2 = 0.9855$), 1/10 (type I collagen from C2C12; $R^2 = 0.9855$), 1/2 (bFGF from HDF; $R^2 = 0.9973$), and 1/4 (IGF-1 from C2C12; $R^2 = 0.9729$) for the reliable data within the ELISA sensitivity range (pg mL^{-1} to ng mL^{-1}).

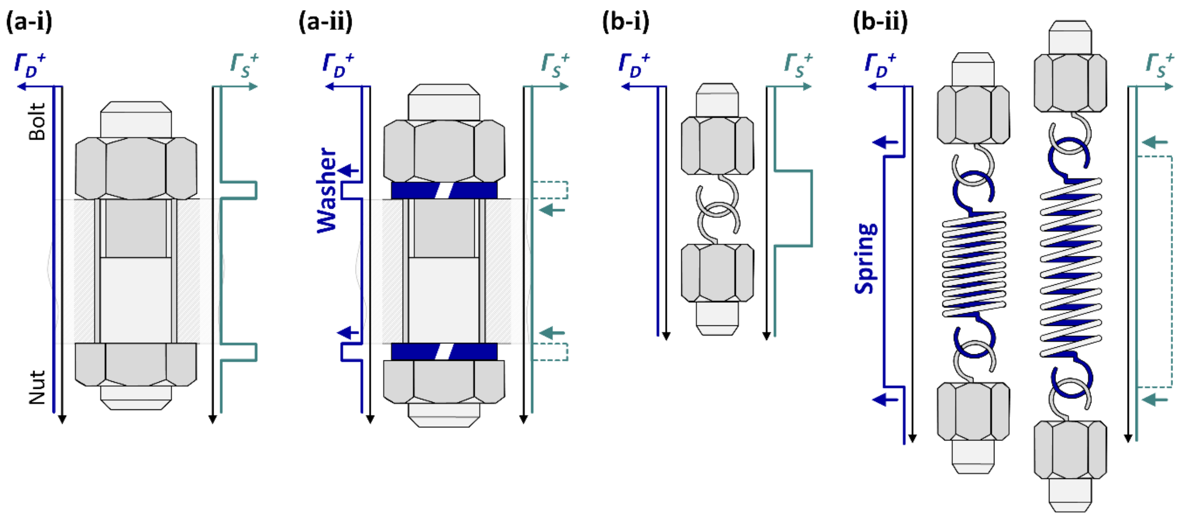

Figure S1. Representative mechanical element organizations comprising the versatile machines. (a-i) Bolt-nut tightening and (b-i) hook connection system without the stress dissipative element hardly suppress the fatigue accumulation. The stress dissipative elements, *e.g.,* (a-ii) washer and (b-ii) spring, were introduced to increase the degree of stress dissipation (Γ_D, blue) and decrease the concentrated stress (Γ_S, green). (a) The washer dissipates the interface pressure at the bolted joint fillet and reduces the false brinelling. (b) The springs scatter the applied stress as a function of the equivalent spring constant when the tensile load is concentrated at the hook connection.

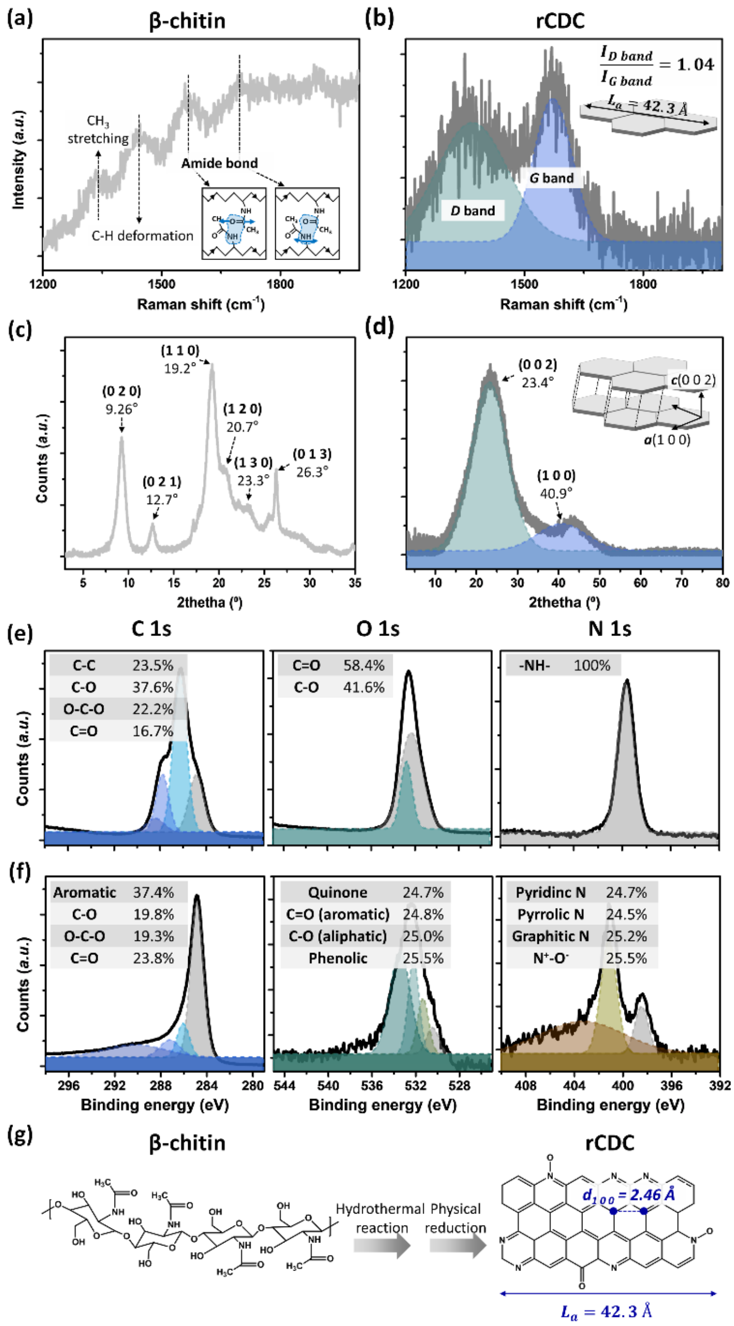

Figure S2. Characterizations of the synthesized electrical filler, so-called physically reduced β-chitin-derived-carbon (rCDC). Raman spectra of (a) β-chitin, (b) rCDC. (a) Raman spectrum of β-chitin was in accordance with previous studies.[3] (b) Near 1.0 value of D/G ratio ($I_{D\ band}/I_{G\ band}$ = 1.04) suggested that the physical reduction resulted in the highly reactive carbon structure.[4, 5] XRD monitorings of (c) β-chitin, (d) rCDC. (c) XRD patterns of β-chitin were observed.[3] (d) (002), (100) peaks suggested the formation of sp^2-hybridized carbon hexagonal structure within rCDC.[6] XPS spectra of (e) β-chitin, (f) rCDC. (e) In the C

1s spectrum of β-chitin, 284.4 eV, 286.2 eV, 287.8 eV, and 288.4 eV corresponded to the C-C (23.5 mol%), C-O (37.6 mol%), O-C-O (22.2 mol%), and C=O (16.7 mol%), respectively. In the O 1s spectrum, 533.2 eV, 533 eV individually suggested the C=O (58.4 mol%), C-O (41.6 mol%). Further, 399.6 eV is accorded with the –NH- of β-chitin. (f) In the C 1s spectrum of rCDC, 284.5 eV, 286.5 eV, 287.8 eV, and 289.5 eV corresponded to the aromatic ring (37.4 mol%), C-O (19.8 mol%), O-C-O (19.3 mol%), and C=O (23.8 mol%), respectively.[7] In the O 1s spectrum, 530.3 eV, 531.4 eV, 532.4 eV, and 533.4 eV individually suggested the quinone (24.7 mol%), C=O in the aromatic ring (24.8 mol%), C-O in the aliphatic chain (25.0 mol%), and the phenol (25.5 mol%).[8] Further, 398.2 eV, 399.5 eV, 400.6 eV, and 403.2 eV accorded with the pyridinc (24.7 mol%), pyrrolic (24.5 mol%), graphitic (25.2 mol%), and oxidizied (N^+-O^--, 25.5 mol%) nitrogen atom, respectively.[9, 10] (e) The schemes suggested that the hydrothermal-carbonization and physical reduction of β-chitin formed the electrical filler (rCDC). The chemical structure of rCDC was conjectured from the three spectroscopies above.

In Figure S2g, the crystallographic characterization of electrical filler (rCDC) was featured according to the D/G ratio (Figure S2b) and XRD pattern (Figure S2d). Following Equation S1, the crystallite size (L_a) was determined as 42.3 Å.

$$I_G/I_G = C(\lambda)/L_a \quad \cdots (S1)$$

here, $C(\lambda)$ (*ca.* 44 Å) was the prefactor of the 532 nm laser for the Raman scattering. Then the d-spacing (d) along with the lattice directions were measured using Bragg's law (Equation S2); that is, $c(002)$ = 3.80 Å and $a(100)$ = 2.46 Å.

$$n\lambda = 2dsin\theta \quad \cdots (S2)$$

here, n was the order of reflection; λ is the wavelength of the incident X-rays; θ was the angle of incidence. In our experimental environment, $n\lambda$ was 1.54.

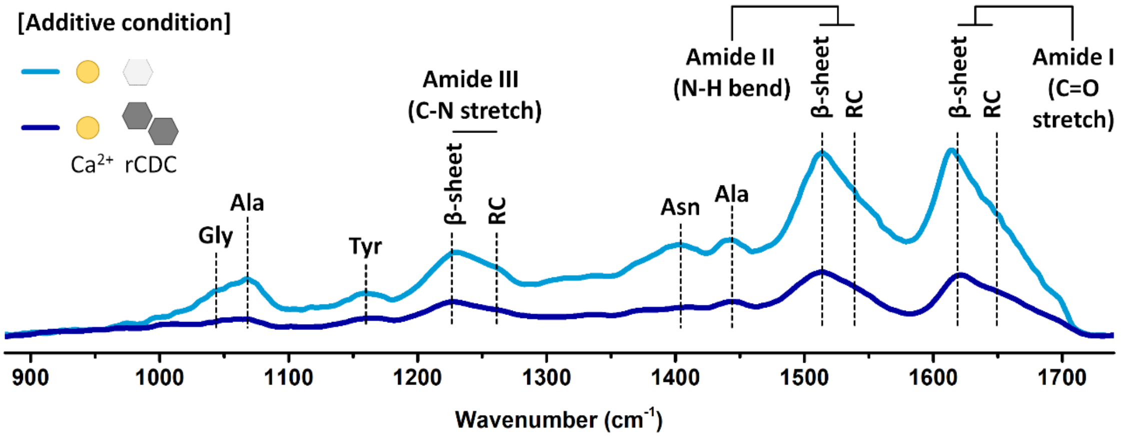

Figure S3. Vibrational characterizations of silk fibroin electrodes. The vibrational properties of amino acids (namely, stretching and bending) were monitored *via* Fourier transform infrared spectroscopy. Yellow circle (○) and black hexagon (⬡) indicates the divalent cation (Ca^{2+}) and rCDC, respectively. Regardless of additive conditions, the representative peaks of silk fibroin were observed; *i.e.,* Gly (~1035 cm^{-1}), Ala (~1080, ~1440 cm^{-1}), Tyr (~1165 cm^{-1}), amide III (~1230, ~1260 cm^{-1}), Asn (~1400 cm^{-1}), amide II (~1505, ~1525 cm^{-1}), and amide I (~1620, ~1650 cm^{-1}).

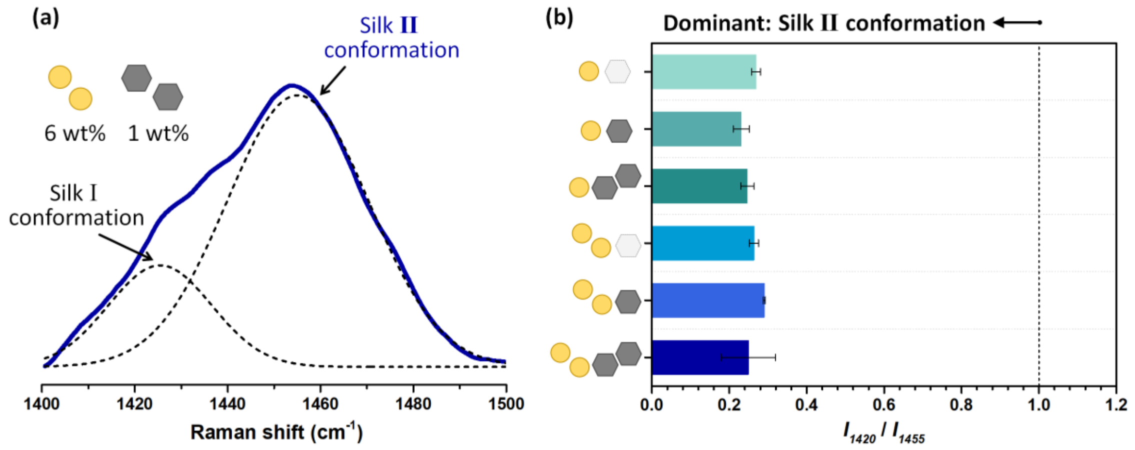

Figure S4. Raman studies to figure out the predominant conformation of silk fibroin electrodes. (a) Representative Raman spectrum (1400 to 1500 cm^{-1}) involves the Raman markers related to the silk I (~1420 cm^{-1}) and II (~1455 cm^{-1}) conformation. (b) The processed I_{1420}/I_{1455} values were below 1.0, indicating the silk II conformation was dominant regardless of the additive condition. [11]

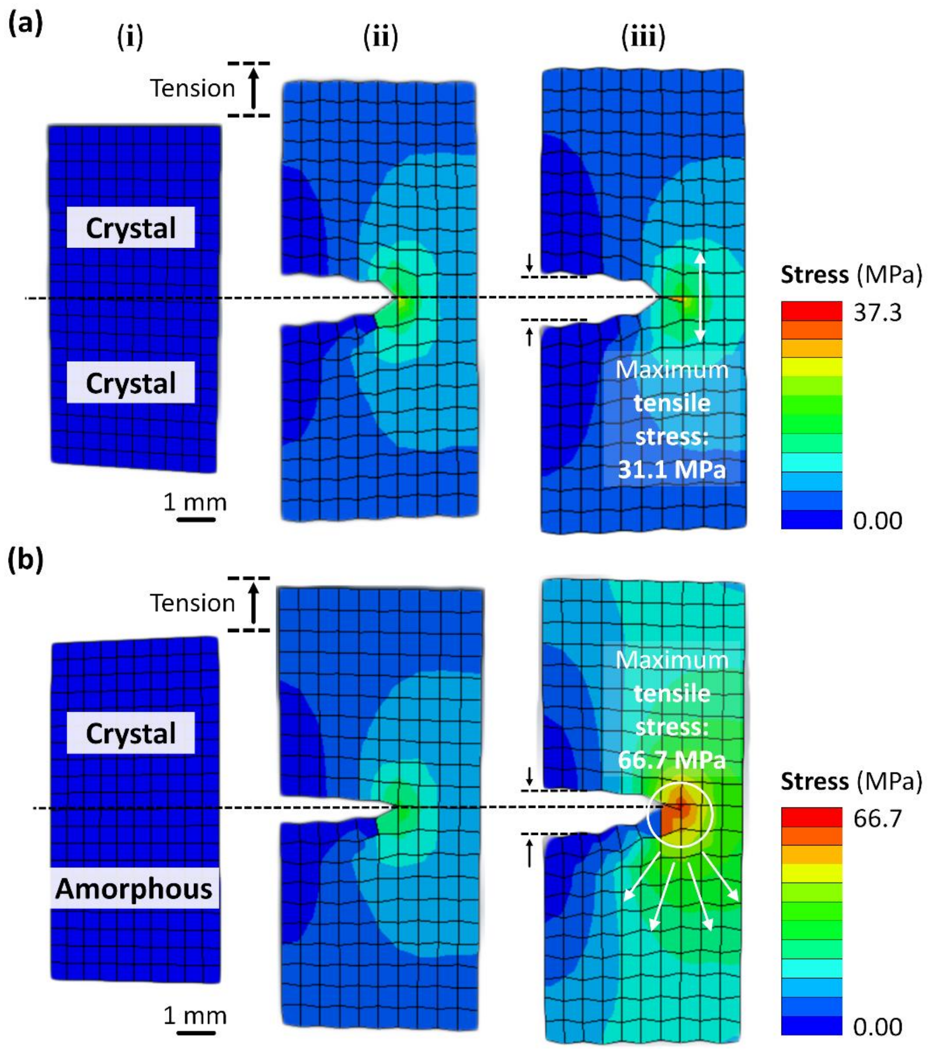

Figure S5. Extended finite element simulation of the modeled silk fibroin beam. (a) Symmetric (crystal-crack-crystal), (b) asymmetric (crystal-crack-amorphous) beam model were subject to the constant tensile load (*i.e.,* 30 MPa). The initial aspect ratio of beam models was 1.86. The stress distribution and crack propagation within the beams were tracked in (i) initial, (ii) intermediate, and (iii) final state. When the crack propagation was reached ~70% of the crack axis, the simulation was saturated. The characteristics of secondary structures and details about simulation were summarized in Table S1.[12]

The extended finite element simulation (here, the tyrosine crosslink was off the table) further evidenced the essential role of interfacial tyrosine crosslink in Γ_D. Figure S5 and Supporting Video S1 and S2 inform that the *in situ* crack propagations resulted from the tensile stress. According to the symmetric coarse-grained model (*i.e.,* all around propagating

crack is crystal domain), the uniformly distributed stress maximally converged to 31.1 MPa at the crack tip. However, the symmetric crystal structure hardly represented the realistic model of the silk fibroin.[13] Therefore, the asymmetric beam was evaluated, in which the crack was flanked by both crystal and amorphous domains. In the asymmetric model, the stress was non-uniformly biased to the amorphous region. Additionally, the amorphous side crack tip underwent significant tensile stress of 66.7 MPa, ~2.14 times enhanced stress of the symmetric model. Without the stress dissipative crosslink of two disparate domains, the delicate amorphous strand (*i.e.,* 9.9 pN $Å^{-1}$) would preferentially be fractured; that is, the stumbling block of elastoplasticity.

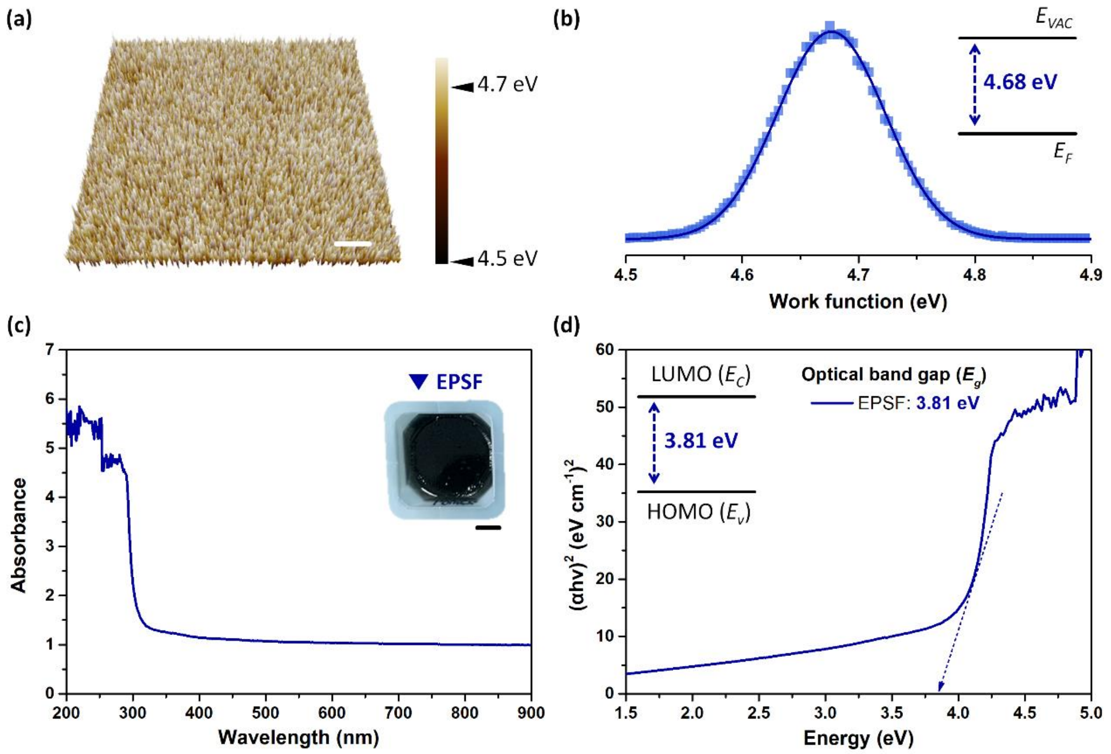

Figure S6. Electron energy level of silk fibroin electrode. (a-b) The work function, (c-d) optical band gap were measured. (a) KPFM mapping result of EPSF. The scale bars indicate 1.0 µm. (b) Histograms extracted from Figure S6a. The work function was 4.678 eV for EPSF. Here, the work function was defined as the variation between the local vacuum level (E_{VAC}) and Fermi level (E_F).[14] The reference work function of silk fibroin was 4.73 eV.[15] (c) UV-visible absorbance spectra. As summarized in the inserted photograph (scale bar = 1cm), the hardly transparent EPSF resulted in the non-zero converged absorbance. (d) Optical band gap energy was figured out from the Tauc plot.[16] Here, the optical band gap energy denoted the difference between LUMO and HOMO (*i.e.,* the lowest unoccupied or highest occupied molecular orbitals). The optical band gap of EPSF was 3.81 eV.

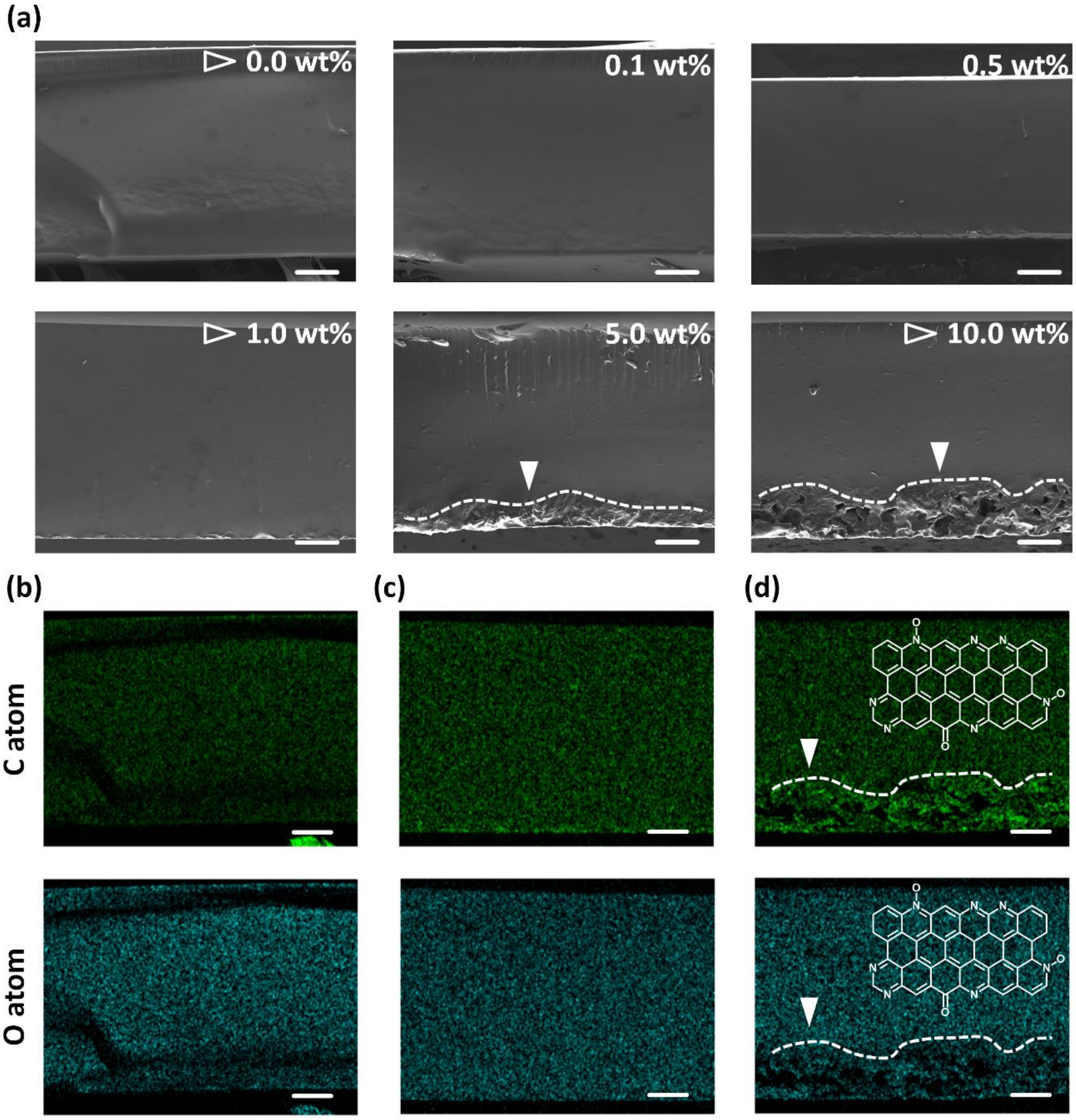

Figure S7. *In situ* evaluation of sedimentation as a function of electrical additive concentration (Φ_{rCDC}). (a) Cross-sectional SEM images. The thickness was fixed at 500 μm regardless of Φ_{rCDC}. The samples with the empty arrows were subjected to atom mapping. (b-d) Cross-sectional EDS images; (b) Φ_{rCDC} = 0.0 wt%, (c) 1.0 wt%, and (d) 10.0 wt%. The solid arrows indicated the precipitation of the electrical fillers was not observed until Φ_{rCDC} = 1.0 wt%. The scale bars indicate 100 μm.

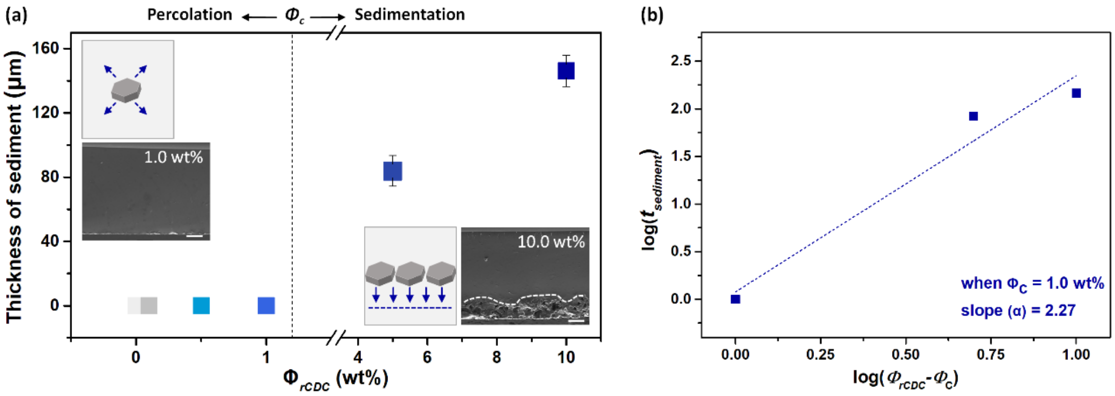

Figure S8. Percolation theory to determine the critical concentration of electrical filler. (a) The thickness of sediment was measured as a function of the Φ_{rCDC} to determine the critical concentration for the percolation (Φ_{c}). The scale bars of inserted SEM images indicate 100 μm. (b) Power law of percolation theory to evaluate Φ_{c}.[17]

The various fillers have been introduced to produce mechanically and electrically high-performance conductors, such as Hofmeister series ions,[18] inorganic nanoparticles,[19] carbon derivatives,[20] and polysaccharides.[21] For instance, 2D carbon elements helped to produce highly conductive biopolymers.[22, 23] However, the introduced fillers may present the gravitational sedimentation and interrupt the polymer strands according to Stoke's law.[24, 25] Since the density of electrical filler (rCDC; ~2.26 g cm^{-3}) was higher than the value of silk fibroin (~1.40 g cm^{-3}), the spontaneous sedimentation should be investigated.

When $\Phi_{rCDC} < \Phi_C$, the introduced fillers solely formed the percolation network. Otherwise, the sedimentation would be spontaneous upper Φ_C. According to the power law in Equation S3 and S4, Φ_C was rationally figured out as a function of sediment thickness ($t_{sediment}$).[17] In Equation S4, α was the index to judge Φ_C.

$$t_{sediment} \propto (\Phi_{rCDC} - \Phi_C)^{\alpha} \quad \cdots \text{(S3)}$$

$$From\ Equation\ \text{(S3)};\ \log(t_{sediment}) \propto \alpha log(\Phi_{rCDC} - \Phi_C) \quad \cdots \text{(S4)}$$

Technically, when the appropriate Φ_C was taken into account, α would be in the range of 1.6 to 2.5. As proof of concept, the linear slope (α) in Figure S8 was 2.27, assuming Φ_C = 1.0 wt%. Therefore, Φ_{rCDC} = 1.0 wt% was the upper limt of percolation threshold.

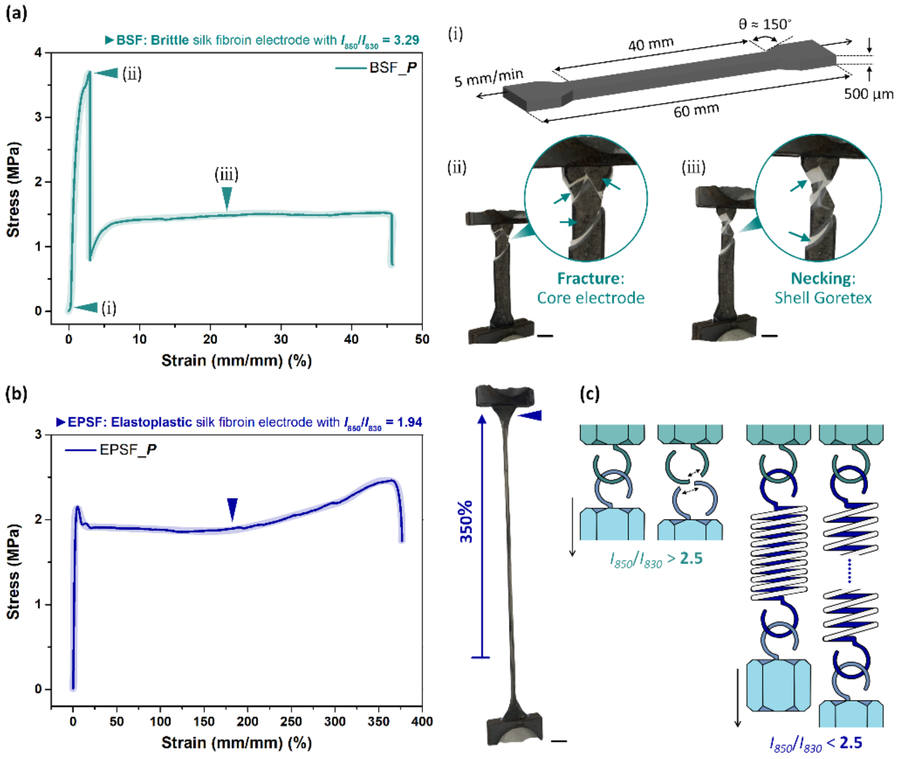

Figure S9. Detailed investigations of engineering strain *vs.* stress curves of core-shell fabric bands. The core electrodes were (a) brittle silk fibroin electrode with $I_{850}/I_{830} = 3.29$ (BSF) and (b) stress dissipation encoded elastoplastic silk fibroin electrode (EPSF; $I_{850}/I_{830} = 1.94$). The photographs during tension were summarized on the right side of the curves. The scale bars of photos indicate 1.0 cm. (a-i) All specimens were processed into the dog bone shapes regarding ISO 527-2 (specimen type 1BA) for the mechanical characterization. (a-ii) The core electrode (BSF) is directly fractured without stretching, meaning the nonharmonic phase separation. (a-iii) The shell Goretex solely resulted in the quasi-stable necking. (b) Stress dissipation encoded silk fibroin electrode led to the superior necking and strain hardening behavior of EPSF fabric band.[26] (c) Illustration depicting the inherent mechanical responses of BSF and EPSF according to the stress dissipative tyrosine crosslink (*i.e.*, spring in hook connection).

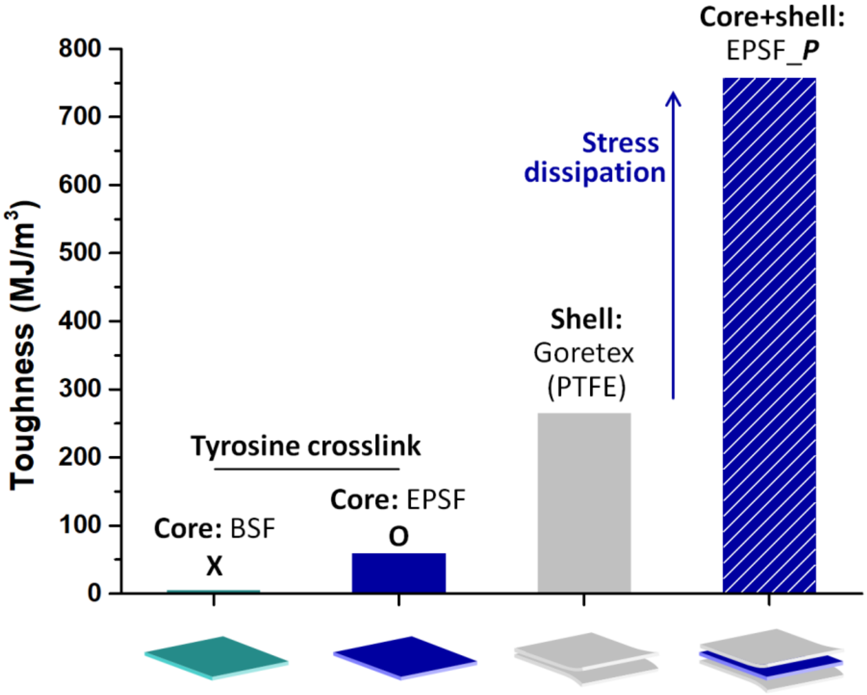

Figure S10. Mechanical strengths of the core electrode and core-shell fabric bands are improved by the stress dissipation of tyrosine crosslink.

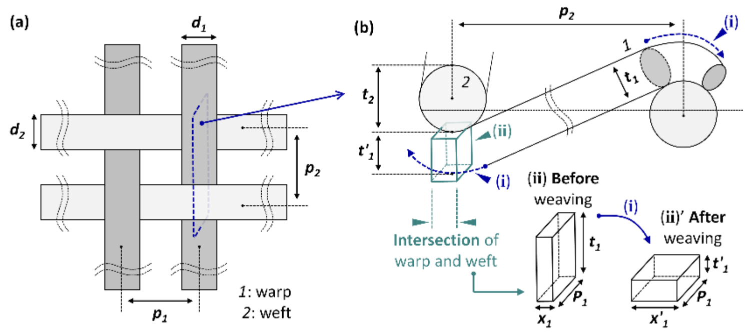

Figure S11. Model study of localized intersection comprising the woven structure. (a-b) The geometry of the plain weave. (a) Top and (b) cross-section view to estimate the localized deformation and stress during the weaving process.[27] Here, suffixes 1 and 2 denoted the warp (EPSF_*P*) and weft (EPSF_*N*), respectively; *d* was the warp (or weft) diameter; *p* was the warp (or weft) spacing; *t* was the warp (or weft) thickness; *x* was the width of unit intersection cell (green hexahedron cube). Superscripts (') suggested the flattened warp (or weft) due to the weaving.

When weaving the fabric with near-unrealistic construction, the cloth's fell would creep beyond the reed's forward position, and the bumping would occur.[27] The coverage factor (*k*) was the weavability index to predetermine whether the realistic or unrealistic fabric construction. Peirce's geometric parameters of the plain weave were summarized in Figure S11. In the suggested model, Equation S5 should be satisfied to be the weavable structure.[27]

$$\sqrt{1-\left[\frac{28}{(1+\beta)k_1}\right]^2}+\sqrt{1-\left[\frac{28\beta}{(1+\beta)k_2}\right]^2}=1 \quad \cdots (S5)$$

where $k_1 = 28d_1/p_1$, $k_2 = 28d_2/p_2$, and $\beta = t_2/t_1$. The variation of thickness (β) was computed as 3.23, indicating the unit intersection should be deformed substantially for the sustainable weaving. In detail, the unit cell underwent 300% stretching maximally in the warp (or weft) axis to be weavable. The corresponding deformation stress was localized ~150 MJ m^{-3}. The estimated stress was the function of workouts dynamicity and could reach up to 450 MJ m^{-3}.[27]

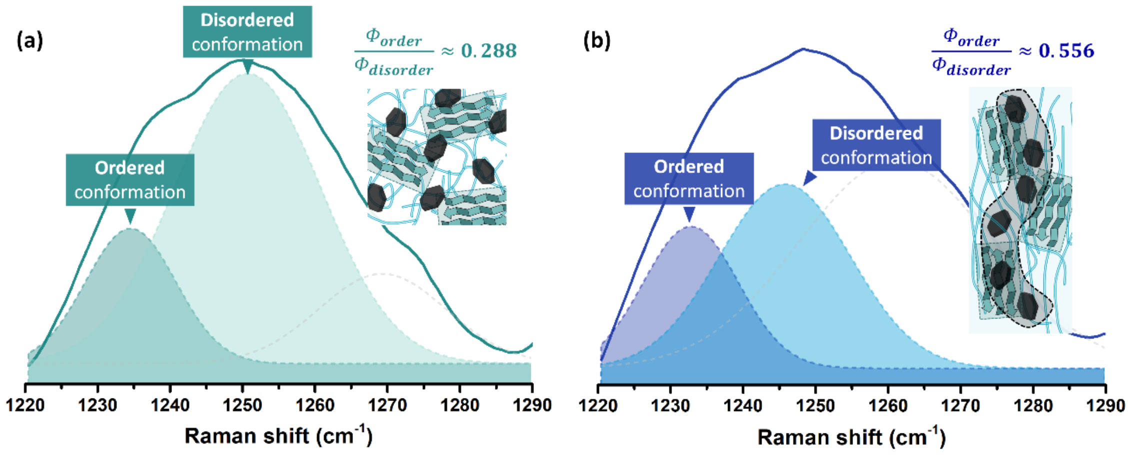

Figure S12. Enhanced intrinsic orders of EPSF according to the tensile load. Raman studies were conducted to elicit the order of crystal from amide III bands when (a) no tension, (b) 130% tensile strain was applied. The Raman marker (*ca.* 1235 cm^{-1}) represented the ordered crystal (*i.e.*, Φ_{order}). Otherwise, the Raman components (*ca.* 1250 cm^{-1}) corresponded to the disordered random coil conformation (*i.e.*, $\Phi_{distort}$).[11, 28] As the tensile strain reached 130%, the order of intrinsic structures simultaneously increased ~193%.[23]

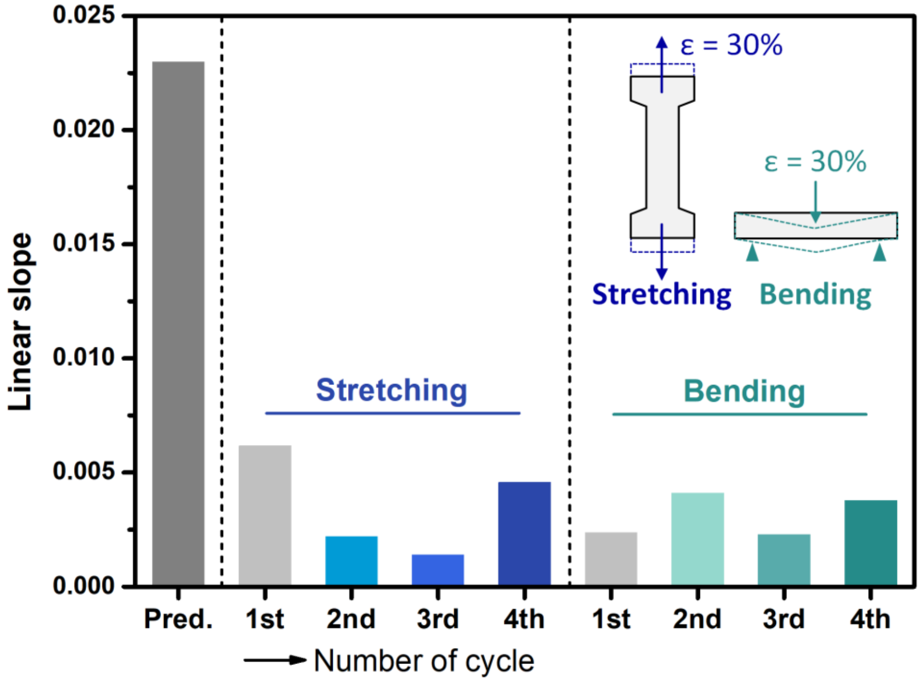

Figure S13. Stable electromechanical response of EPSF under various loads. The linear slope of R/R_0 in Figure 4e was estimated. *Pred.* (*i.e.,* predicted) was calculated from Pouillet's law.

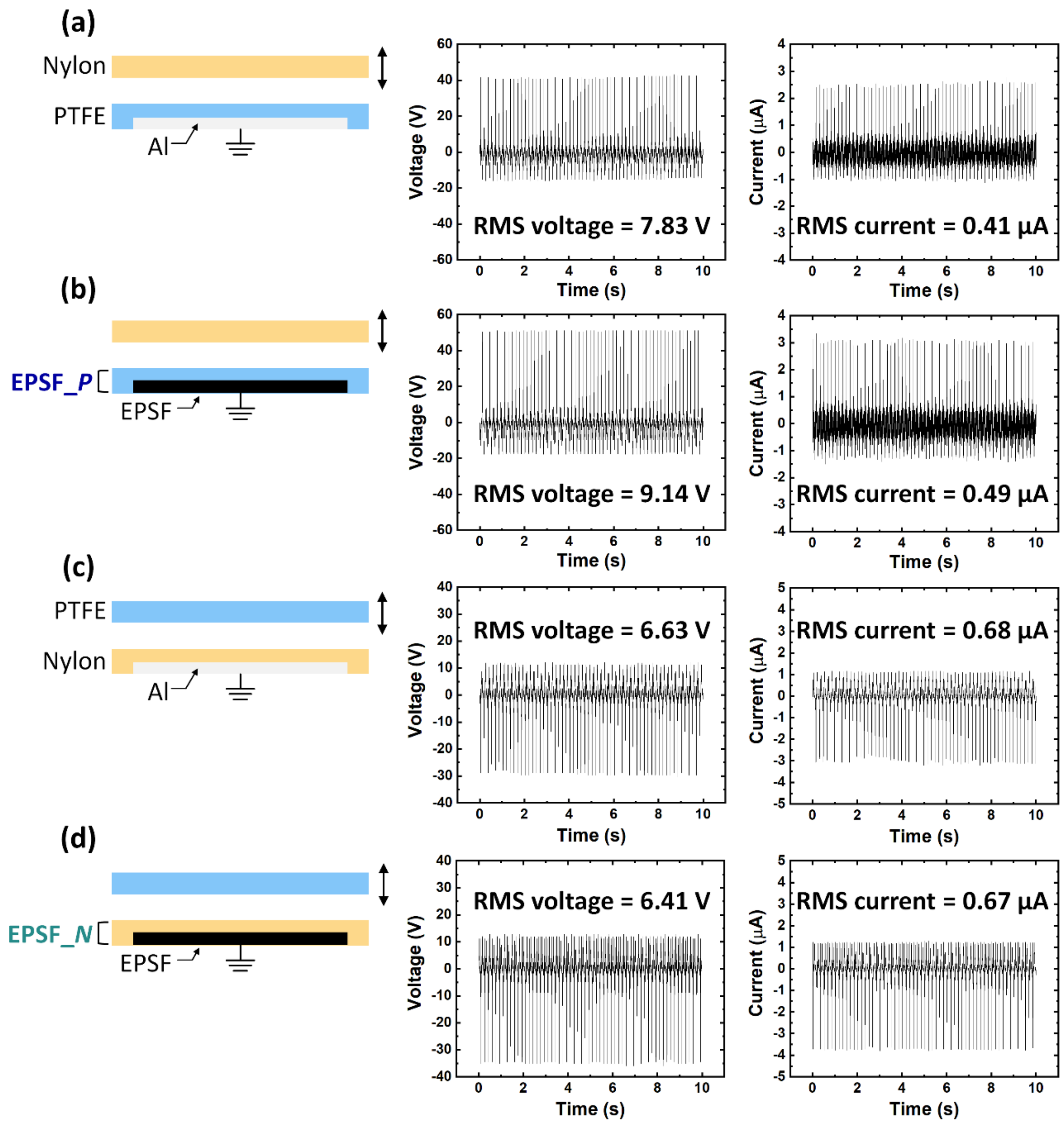

Figure S14. Investigation of electrical output *via* diverse single electrode modes.

Here, the suitability of EPSF for triboelectrification was inquired. The single electrode mode was adopted as general triboelectrification-based electricity generation method. Figure S14 represented the electrical outputs (*i.e.,* peak open-circuit voltage (V_{OC}) and peak closed-circuit current (I_{CC})) of single electrode mode in various conformations. Briefly, the electrodes (aluminum (Al) or EPSF) were incorporated with the dielectric polytetrafluoroethylene (PTFE) or nylon fabric bands. Each PTFE and nylon is representative negative and positive triboelectric material. The pre-synthesized EPSF fabric band underwent vertical contact and separation in 6 Hz with the counterpart dielectric fabric band. Thus, the

continual electrical output was generated *via* the triboelectrification between fabric bands and electrostatic induction. The root mean square (RMS) voltage and current calibrated the irregular peak waveforms following Equation S6.

$$RMS\ voltage = \sqrt{\frac{\int V^2(t)dt}{T}}, RMS\ current = \sqrt{\frac{\int I^2(t)dt}{T}} \quad \cdots (S6)$$

where each $V(t)$, $I(t)$, and T represents measured peak voltage output, peak current output, and measurement period.

Regardless of the polarity of surface charge (representatively, polar directions of peak outputs), Al and EPSF generated comparable electrical performances, suggesting that EPSF could behave as the electrode similar to Al. In Figure S14a, Al has produced a maximum peak V_{OC} of 43.2 V (RMS 7.83 V) and peak I_{CC} of 2.8 μA (RMS 0.41 μA). In Figure S14b, EPSF has generated a maximum peak V_{OC} of 51.2 V (RMS 9.14 V) and a peak I_{CC} of 3.3 μA (RMS 0.49 μA). The opposite case was further studied; *i.e.,* the electrodes were incorporated with nylon, and PTFE acted as the counterpart. In this conformation, Al has generated a maximum peak V_{OC} of 30.4 V (RMS 6.63 V) and a peak I_{CC} of 3.2 μA (RMS 0.68 μA) (Figure S14c). EPSF has produced a maximum peak V_{OC} of 36 V (RMS 6.41 V) and a peak I_{CC} of 3.8 μA (RMS 0.67 μA) (Figure S14d).

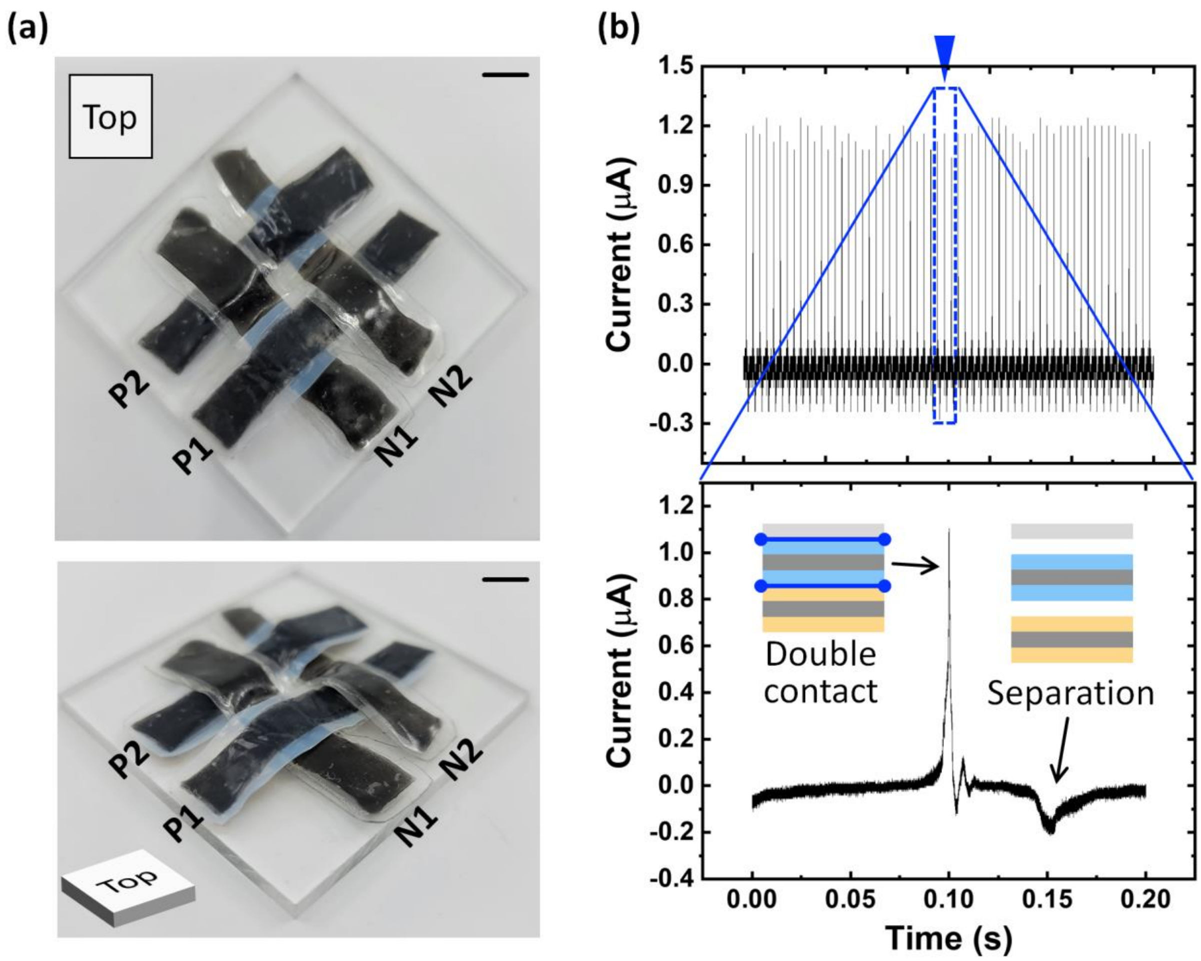

Figure S15. 2 × 2 fabric band unit for the detailed investigation of the realistic woven structure. (a) Photograph of 2 × 2 unit. P and N means PTFE and nylon, respectively. The scale bar in Figure S15a indicates 1 cm. (b) Peak I_{CC} output plot of 2 × 2 unit. The lower corresponds to the magnified I_{CC} peak. The light gray, blue, yellow, and dark gray indicate cotton, PTFE, EPSF, and nylon.

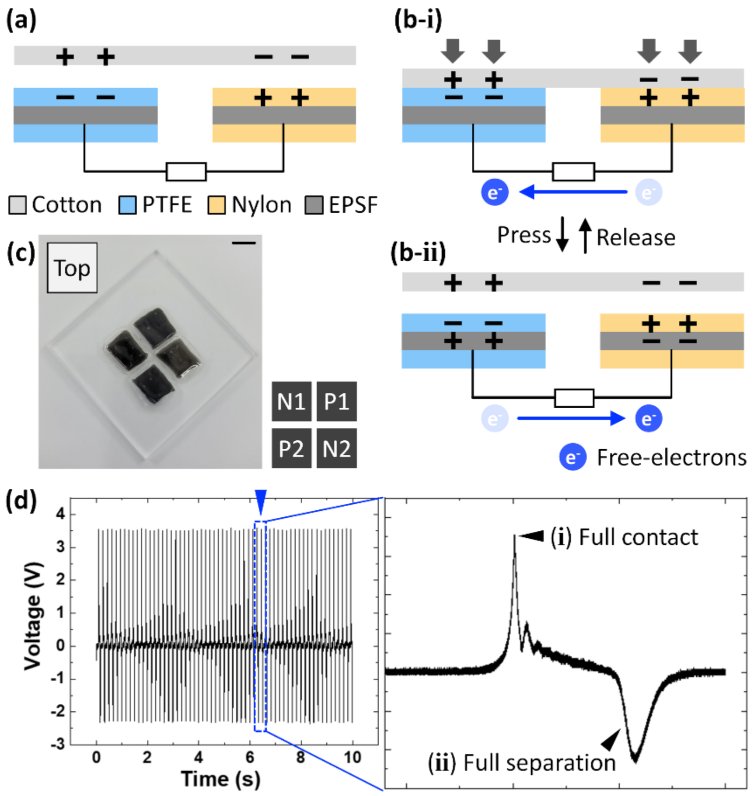

Figure S16. A simplified interpretation of woven-based triboelectrification (a-b) Reduced working mechanism. (c) Representative photograph and (d) Peak V_{OC} plot of supposed single contact mechanism. The scale bar in Figure S16c indicates 1 cm.

Previous studies on triboelectric fabric or textile hardly represented the woven structure, and the simplified working mechanism was proposed as summarized in Figure S16a.[29] In other words, a slightly nonrealistic mechanism, considering only single contact with cotton, was featured; that is, a small number of free-electrons flowed due to the no additional surface charges by internal contact within the woven structure (Figure S16b). The electrical output of the model device in Figure S16c was measured. In Figure S16d, the single contact mechanism sample generated a maximum peak V_{OC} of 3.6 V. Notably, the maximum peak V_{OC} of a single contact mechanism was 6.5 times lower than V_{OC} of double contact mechanism.

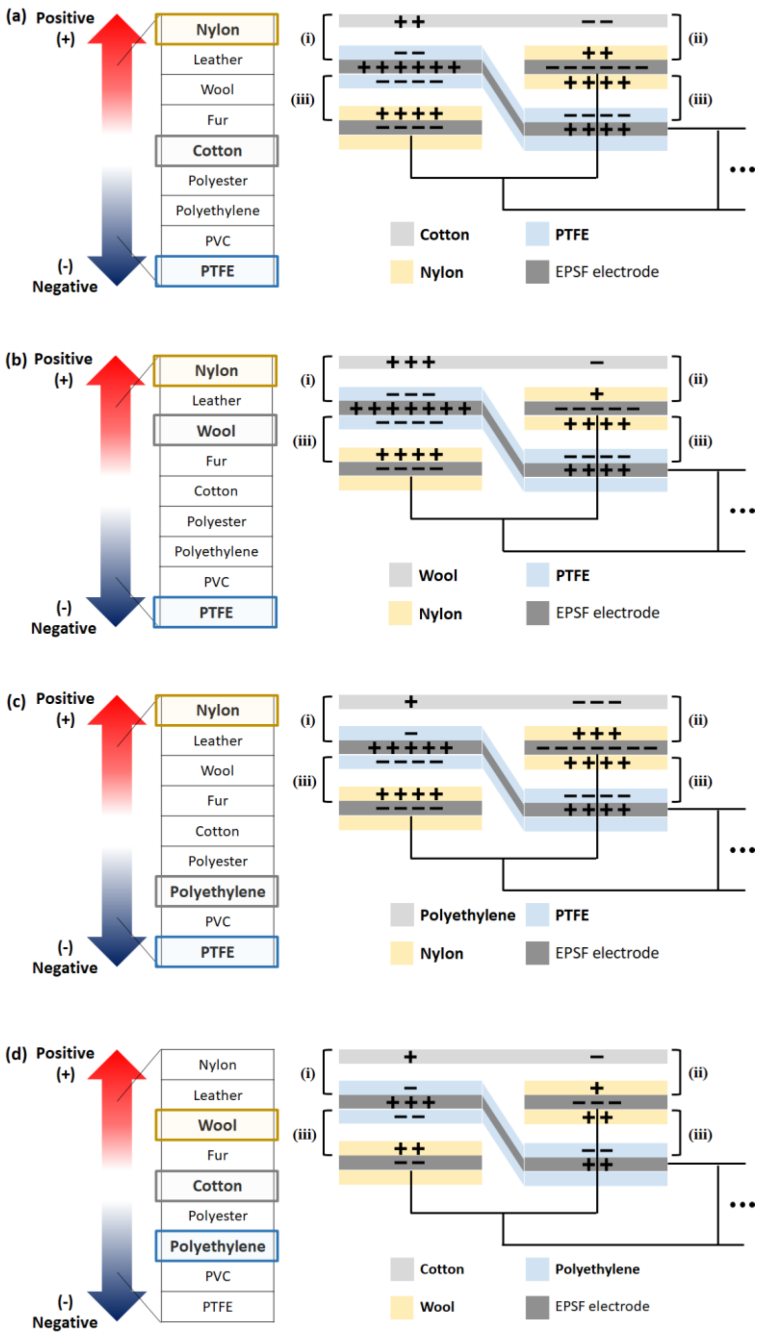

Figure S17. Various material selection possibilities in fabric-related triboelectric series and corresponding electrical mechanisms. (i-ii) and (iii) indicates the external and internal contact, respectively.

In this work, among fabric-related triboelectric series, each PTFE (*i.e.*, Gore-tex) and Nylon was adopted for packaging EPSFs as the most negative and positive material. Owing to the large differences in electron affinities, the packaged EPSFs can be more efficiently charged *via* (iii) internal contact with each other (Figure S17a). Moreover, cotton was used as a

counterpart material for (i, ii) external contact due to its neutral position compared to PTFE and nylon. The possibility of connections is (i) PTFE-cotton, (ii) nylon-cotton, and (iii) PTFE-nylon.

If other slightly positive fabric-related triboelectric material such as wool is considered as counterpart material, there is no significant effect on electrical output performance (Figure S17b). It is because although the amount of charge generation at external contact (ii) reduces, the charge generation amount at external contact (i) increases, and thus total charge generation amount remains similar to that of Figure S17a. The possibility of connections is (i) PTFE-wool, (ii) nylon-wool.

The tendency above will be identical for polyethylene as counterpart material which is a slightly negative material; in other words, decreased surface charges at external contact (i) are compensated by external contact (ii) (Figure S17c). The possibility of connections is (i) PTFE-polyethylene, (ii) nylon- polyethylene.

Meanwhile, suppose other fabric-related triboelectric materials such as polyethylene and wool are exploited to package EPSFs instead of PTFE and nylon respectively when the counterpart material is cotton (Figure S17d). In that case, the total amount of surface charges will decrease during both external (i, ii) and internal contact (iii) because the relative difference of electron affinity becomes small between each internal and external contact material. Therefore, the small number of free electrons will flow between each EPSF electrode by electrostatic induction. Here, the possibility of connections is (i) polyethylene-cotton, (ii) wool-cotton, (iii) polyethylene-wool.

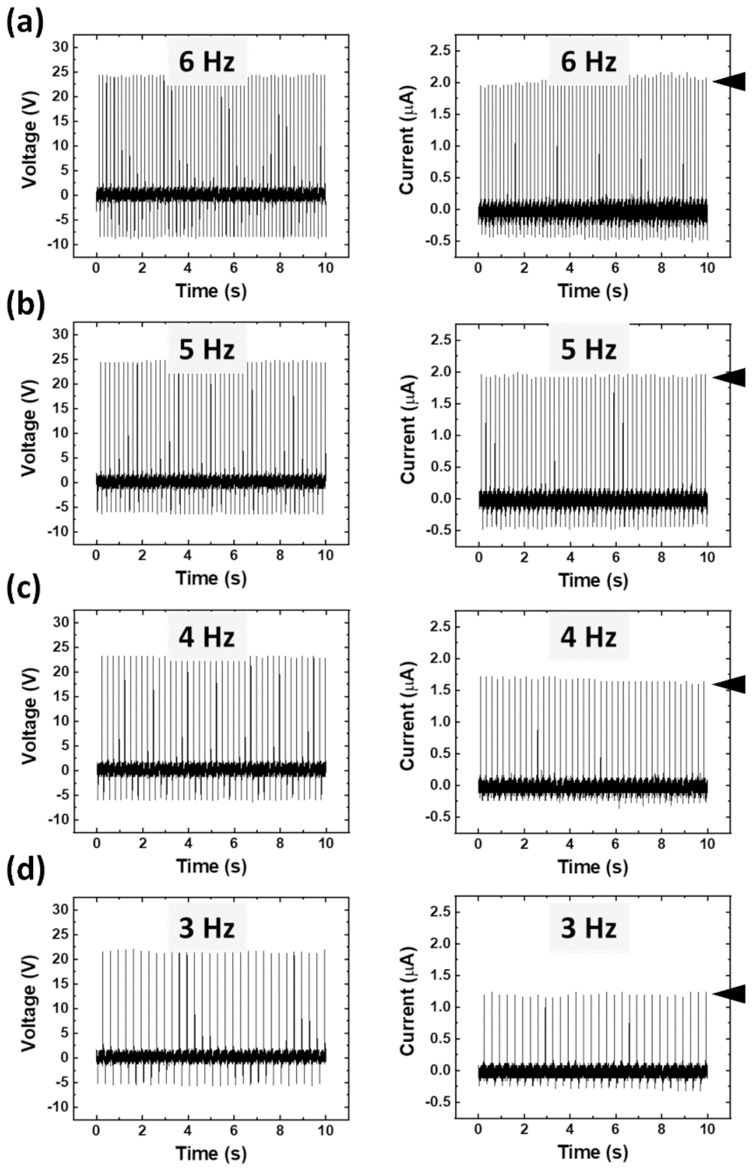

Figure S18. Peak V_{OC} plots of 2 × 2 unit as a function of contact-separation frequency.

During the workouts, various body motions may cause irregular contact-separation frequency. In this regard, the electrical output of 2 × 2 unit was evaluated depending on vertical input frequency (Figure S18). As the frequency increases from 3 Hz to 6 Hz, a similar peak V_{OC} output of *ca.* 23 V was measured (left column in Figure S18). But peak I_{CC} output increased from 1.24 to 2.16 μA (right column in Figure S18), following the previous studies on triboelectrification-based electricity generation.[30, 31]

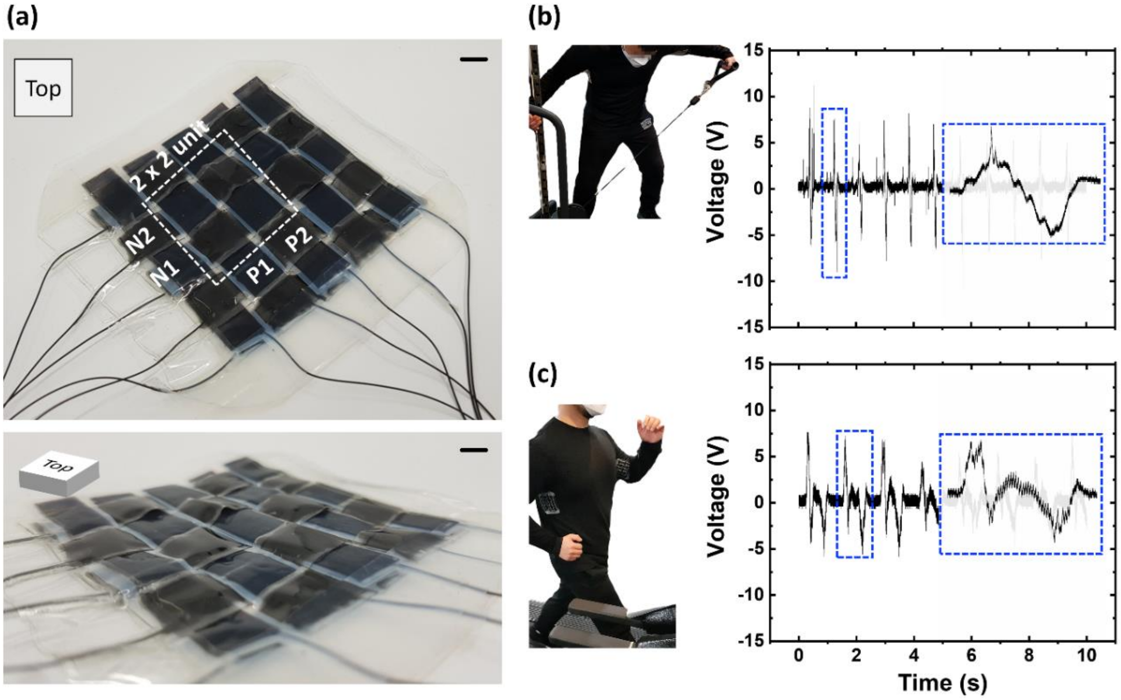

Figure S19. Triboelectric output as the potential power source of EPSF bioelectronics. (a) Representative photograph of 5 × 5 fabric band device. The scale bars indicate 1.0 cm. Voltage outputs (V_{OC}) during (b) vertical and (c) sliding motions. Figure S19b showed the measured V_{OC} outputs (*i.e.,* 11.2 V) when 5 × 5 fabric band device underwent vertical contact with the sportswear (*e.g.,* during cable side lateral raise). In Figure S19c, the sliding contact motion (*e.g.,* during running) has generated the maximum peak V_{OC} of 8.6 V and produced the double peaks originated from the two times of contact-separations during one swaying arm back and forth.

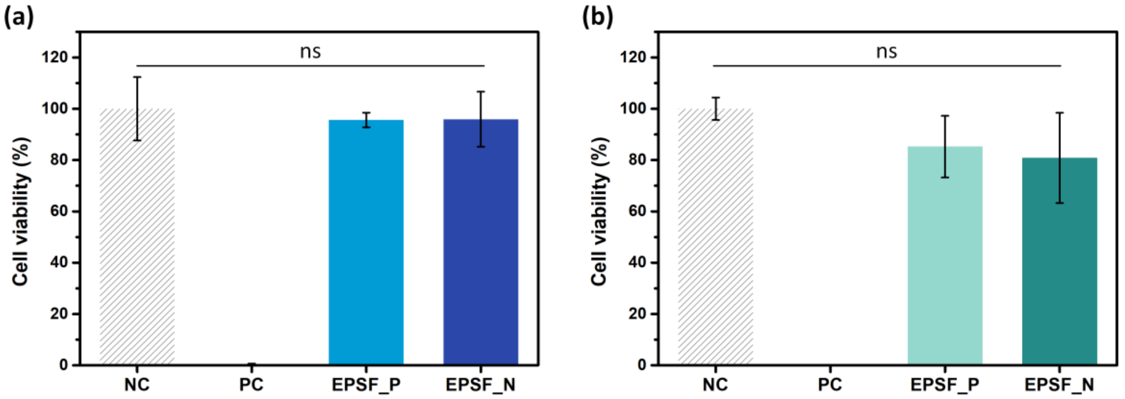

Figure S20. Cytotoxicity of the fabricated fabric bands. EPSF_*P*, EPSF_*N* hardly presented the toxicity to the (a) fibroblast (human dermal fibroblast, HDF), (b) myoblast (C2C12). Here, *NC* and *PC* denoted the negative control (100% alive cells without any process) and the positive control (100% dead cells by 20 vol% DMSO treatment), respectively. The non-significance (*ns*) was elicited from the one-way ANOVA, and *ns* indicated *P*-value > 0.05. The error bars and statistical analysis were originated from n=6 results.

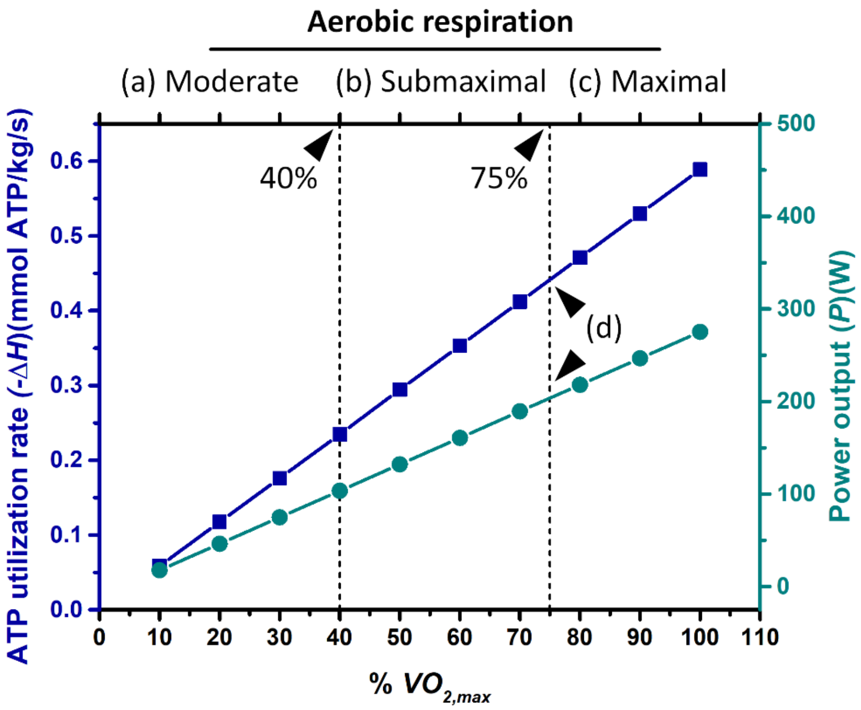

Figure S21. Percentage of maximum oxygen uptake (% $VO_{2,max}$) as the indicator quantifying the workout intensity. Briefly, the moderate, submaximal, and maximal workout corresponded to (a) % $VO_{2,max}$ < 40%, (b) 40% < % $VO_{2,max}$ < 75%, and (c) % $VO_{2,max}$ > 75%, respectively.

The type of sports performance considerably affected the source of ATP supplement.[32] In terms of the sprint events lasting seconds, the dominant ATP was replenished from the anaerobic metabolism (*e.g.,* breakdown of phosphocreatine and glycogen). Otherwise, the aerobic metabolism (*e.g.,* phosphorylation of carbohydrate and fat) supplied the vast ATP for the long-term muscle contraction events lasting hour scale. In detail, the aerobic metabolism solely relied on carbohydrate oxidation to perform the higher intensity, *vice versa*.[32] Since the daily workout routine usually carried on at least one hour, the aerobic metabolism was taken into consideration.

Here, the percentage of maximum oxygen uptake (% $VO_{2,max}$) was the quantified index of workout intensity to conduct the mathematical and theoretical biology (Figure 6b-d). Since the respiratory exchange ratio collected at the mouth presented the metabolic activity at mitochondrion, the regarding work intensity as a function of % $VO_{2,max}$ was one of the general

approaches in the exercise physiology (*e.g.,* spirometry device).[32, 33] The cross-validation procedure was conducted to verify whether % $VO_{2,max}$ represented the workout intensity in the mathematical biology approach.

First of all, the clinical database of the college-aged students was gathered referring to the prior studies (Table S2). Then, the intuitive index, power output (P in watt (W)), was explored in the green line plot (●) of Figure 6c and S21. Here, P indicated the muscular contraction activity performed by specific oxygen uptake (% $VO_{2,max}$). The empirical dependence of the two variables was as the following relationship; % $VO_{2,max}$ = 13.895P+151.[34] The comparative analysis between calculated and clinical P values verified the reliability of the approach above; that is, P(75% $VO_{2,max}$) was 203 W in the mathematical biology (Figure S21d), and 200 W from the clinical measurement.[32] Therefore, the theoretical investigation of % $VO_{2,max}$ accurately informed the workout intensity.

In the blue line plot (■) of Figure S21, the ATP utilization rate to perform the specific intensity of muscle contraction (-ΔH in mmol ATP kg^{-1} s^{-1}) was estimated following Equation S7 and S8.[35]

For the workouts below the maximal intensity $(0\sim75\%\ VO_{2,max})$;

$$-\Delta H = (18.56)(\%\ VO_{2,max}) + (2.40)(\%\ VCO_{2,max}) - 4.14N \quad \cdots (S7)$$

For the maximal workout $(75\sim100\%\ VO_{2,max})$;

$$-\Delta H = (18.71)(\%\ VO_{2,max}) + (2.30)(\%\ VCO_{2,max}) - 4.14N \quad \cdots (S8)$$

where % $VO_{2,max}$, % $VCO_{2,max}$ were originated from Table S2. Further, the exhaled nitrogen (N in gram) was a constant value according to the mass balance, *e.g.,* the nitrogen content in the exhalation was consistent with 0.7903 in the Haldane transformation. The computed values were substantially dependable since the mathematics was accorded with the clinics; that is, -ΔH (*75%* $VO_{2,max}$) was 0.442 mmol ATP^{-1} kg^{-1} in Figure S21d and 0.4 mmol ATP^{-1} kg^{-1} in the

clinical results.[32] Therefore, the vast ATP (*i.e.,* higher -ΔH) was required as the muscle activity enhanced (*i.e.,* higher % $VO_{2,max}$).

The first compensation of the required ATP resynthesis (-ΔH) was the consumption of intramuscular ATP storage.[32] The general intramuscular ATP storage was not sufficient, *i.e.,* 5 mmol kg^{-1}. Therefore, ATP stock would be exhausted within 15 s to perform the submaximal muscle contraction event since -ΔH(75% $VO_{2,max}$) was 0.442 mmol ATP kg^{-1} s^{-1}. Figure 6c was obtained regarding ATP storage would be positively expanded as the cellular activity improved (Figure S22).

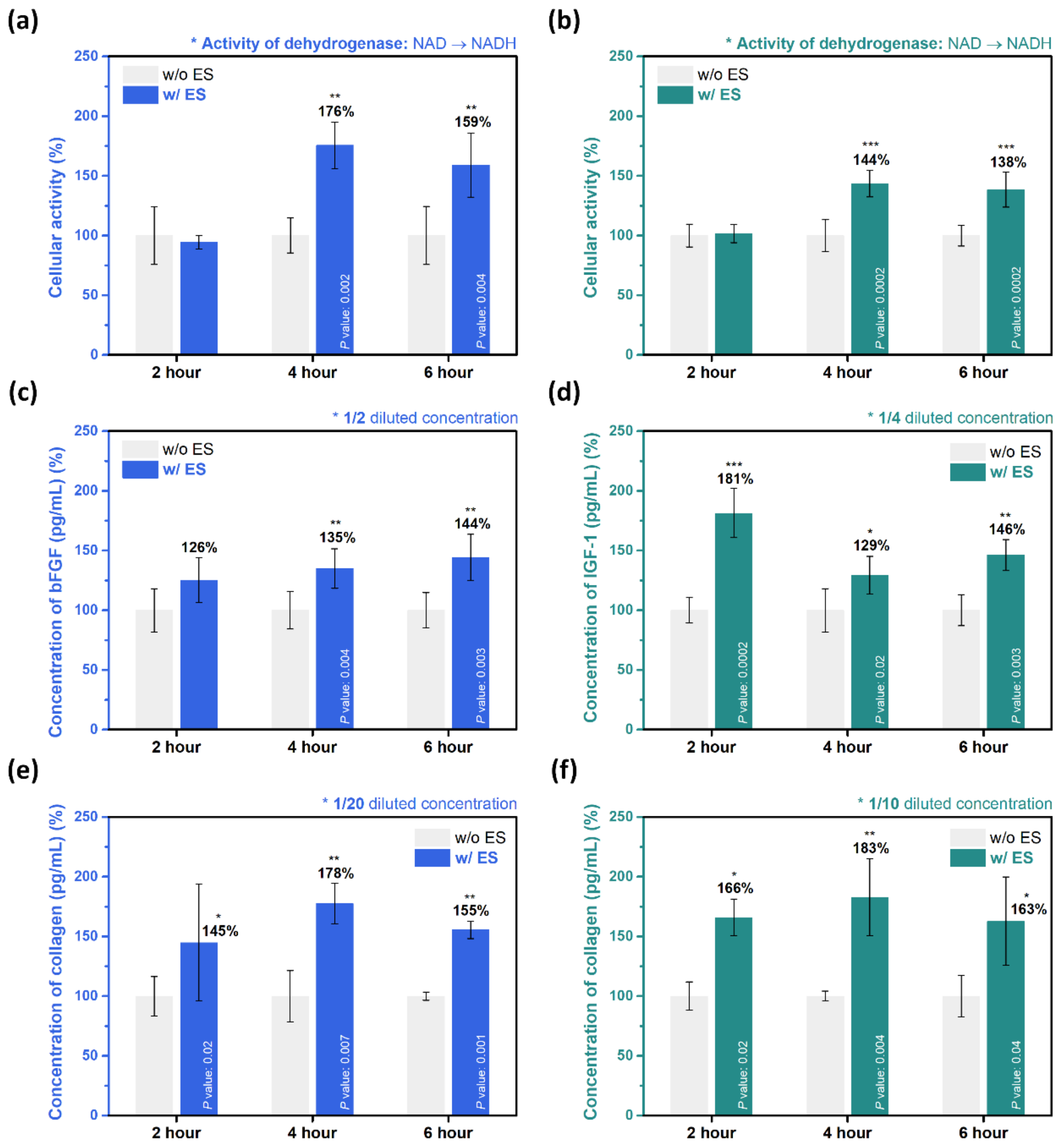

Figure S22. *In vitro* electrostimulation of model fibroblast and myoblast. The model cells were subjected to electrostimulation (ES) for 2, 4, and 6 hours. Here, the power source of ES was the triboelectricity from vertically oscillating 5 × 5 fabric band device. The results involving the blue and green columns were obtained from the fibroblast (human dermal fibroblast; HDF) and myoblast (C2C12), respectively. The light gray columns corresponded to the model cells without ES, *i.e.,* the control group. The numeric values at the end of the color columns suggested the upregulation percentage versus the control group within a specific ES duration. (a-b) The enhanced dehydrogenation rate positively correlated to cellular activities. In metabolism, the dehydrogenase is concerned with the redox-based electron transfer and

enzyme functionality. (c-d) The amounts of growth factors, vital in aerobic metabolism, were evaluated; that is, (c) basic fibroblast growth factor (bFGF) from HDF and (d) insulin-like growth factor (IGF-1) from C2C12. The synthesis enhanced at least 130% since the effective ES activating the voltage-gated plasma channels. (e-f) The synthesis of type I collagen from fibroblast and myoblast was remarkably elevated, indicating the affirmative role of ES in cellular activity. Generally, *in vitro* stimulation efficiency was ranged from 130 to 180%. The color columns were subjected to the Student's test versus the control group for each ES duration. The symbols * (P value < 0.05), ** (P value < 0.01), and *** (P value < 0.001) indicated the statistical significance. The error bars and statistical analysis were originated from n=3 results. *In vitro* experiments were designed since theoretical biology has assumed that the cellular metabolism could represent the whole-body event. Notice that the electroceutical devices (*e.g.,* functional electrical stimulation to induce muscle contraction[36]) have clinically influenced human activity.

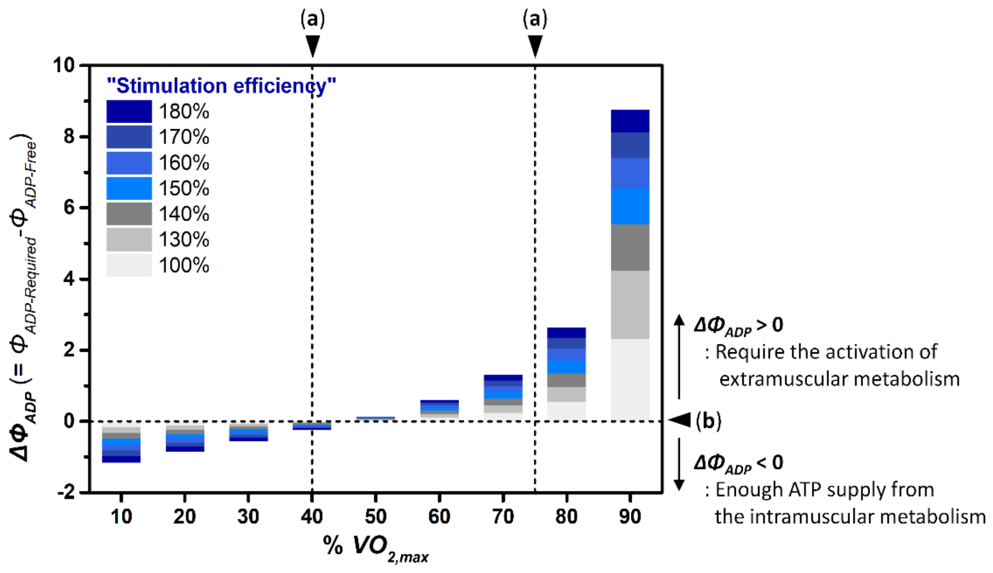

Figure S23. The difference between free and required ADP concentration ($\Delta\Phi_{ADP}$) as a function of workout intensity (% $VO_{2,max}$). (a) Representative % $VO_{2,max}$ sorted the moderate, submaximal, and maximal workout. The relationship between workout intensity and % $VO_{2,max}$ corresponded to Figure S21. (b) The boundary condition of $\Delta\Phi_{ADP}$; *i.e.,* $\Delta\Phi_{ADP} = 0$ indicated whether the extramuscular metabolism should be activated to replenish the deficient ATP.

In Equation S9 and S10, the required ADP concentration to perform the specific muscle contraction ($\Phi_{ADP\text{-}Required}$) was deduced from Michaels-Menten kinetics (middle term in Equation S9) and spirometry principal (right term in Equation S9).[37]

$$VO_{2,op} = \frac{VO_{2,max}}{1 + K_{s1}/\Phi^2_{ADP-Required}} = \frac{(P)(K_{s4}) + (W)(VO_2)}{W} \quad \cdots (S9)$$

$$\Phi^2_{ADP-Required} = \frac{(K_{s1})[(P)(K_{s4}) + (W)(VO_2)]}{(W)(VO_{2,max}) - (P)(K_{s4}) - (W)(VO_2)} \quad \cdots (S10)$$

where $VO_{2,op}$ was the activity of phosphorylation; K_{S1} was the 50% activity constant of phosphorylation (*i.e.,* 0.0631 $mmol^2\ kg^{-2}$); P, W, and $VO_{2,max}$ was the power output, the body weight, and maximum oxygen uptake, respectively (Figure S21, Table S2). K_{s4} was the oxygen-workload constant. $\Phi_{ADP\text{-}Required}$ was the function of K_{s4} since the respiratory exchange ratio collected at the mouth represented the mitochondrion activity.[33]

Table S1. Parameters for the extended finite simulation. The mechanical properties of silk fibroin were categorized.[12]

**Parameters**	**Crystal domain**	**Amorphous domain**
**Young's modulus**	830 MPa	310 MPa
**Poisson ratio**	0.3	
**Displacement after fracture**	0.4 mm	
**Crack length**	2.0 mm	
**Mesh size**	0.06 m^2	
**Stabilization**	Dissipated energy fraction (0.0002)	
**Maximum stress at fracture**	300 MPa	100 MPa
**In-plane tensile stress (surface traction)**	30 MPa	
	**Symmetric model**	**Asymmetric model**
**Maximum principal stress**	37.3 MPa	66.7 MPa

Table S2. Clinical parameters utilized in Figure 6b-d.[35]

**Subjects**	Untrained college-aged students (n=16)			
**Average body weight**	75 kg			
**Parameters**	**Rest**	**40% $VO_{2,max}$**	**75% $VO_{2,max}$**	**$VO_{2,max}$**
**VO_2** (mL kg^{-1} min^{-1})	4.0	24	38	[a] 53
**VCO_2** (mL kg^{-1} min^{-1})	5.0	32	48	48

[a] Cross validation of the reference values; $VO_{2,max}$ of the college-aged students (n=25) was *ca.* 48 mL kg^{-1} min^{-1}.[38]

References of Supporting Information

[1] D.N. Rockwood, R.C. Preda, T. Yücel, X. Wang, M.L. Lovett, D.L. Kaplan, *Nat. Protoc.*, **2011**, *6,* 1612.

[2] D. López Barreiro, Z. Martín-Moldes, J. Yeo, S. Shen, M.J. Hawker, F.J. Martin-Martinez, D.L. Kaplan, M.J. Buehler, *Adv. Mater.*, **2019**, *31,* 1904720.

[3] M. Kaya, M. Mujtaba, H. Ehrlich, A.M. Salaberria, T. Baran, C.T. Amemiya, R. Galli, L. Akyuz, I. Sargin, J. Labidi, *Carbohydr. Polym.*, **2017**, *176,* 177.

[4] L. Liao, Z. Song, Y. Zhou, H. Wang, Q. Xie, H. Peng, Z. Liu, *Small*, **2013**, *9,* 1348.

[5] J.H. Kim, Y. Choi, J. Kang, E. Choi, S.E. Choi, O. Kwon, D.W. Kim, *J. Membr. Sci.*, **2020**, *612,* 118454.

[6] S.Y. Cho, Y.S. Yun, S. Lee, D. Jang, K.-Y. Park, J.K. Kim, B.H. Kim, K. Kang, D.L. Kaplan, H.-J. Jin, *Nat. Commun.*, **2015**, *6,* 1.

[7] C. Xu, X. Shi, A. Ji, L. Shi, C. Zhou, Y. Cui, *PLOS ONE*, **2015**, *10,* e0144842.

[8] A. Ganguly, S. Sharma, P. Papakonstantinou, J. Hamilton, *J. Phys. Chem. C*, **2011**, *115,* 17009.

[9] B. Wang, S. Li, X. Wu, J. Liu, J. Chen, *J. Mater. Chem. A*, **2016**, *4,* 11789.

[10] J. Tanum, H. Jeong, J. Heo, M. Choi, K. Park, J. Hong, *Appl. Surf. Sci.*, **2019**, *486,* 452.

[11] P. Taddei, T. Asakura, J. Yao, P. Monti, *Biopolymers: Original Research on Biomolecules*, **2004**, *75,* 314.

[12] A. Nova, S. Keten, N.M. Pugno, A. Redaelli, M.J. Buehler, *Nano letters*, **2010**, *10,* 2626.

[13] S. Keten, Z. Xu, B. Ihle, M.J. Buehler, *Nat. Mater.*, **2010**, *9,* 359.

[14] A. Kahn, *Mater. Horiz.*, **2016**, *3,* 7.

[15] M.K. Hota, M.K. Bera, B. Kundu, S.C. Kundu, C.K. Maiti, *Adv. Funct. Mater.*, **2012**, *22,* 4493.

[16] J.C. Costa, R.J. Taveira, C.F. Lima, A. Mendes, L.M. Santos, *Opt. Mater.*, **2016**, *58,* 51.

[17] F. He, S. Lau, H.L. Chan, J. Fan, *Adv. Mater.*, **2009**, *21,* 710.

[18] N. Mittal, T. Benselfelt, F. Ansari, K. Gordeyeva, S.V. Roth, L. Wågberg, L.D. Söderberg, *Angew. Chem.*, **2019**, *58,* 18562.

[19] C. Kwon, D. Seong, J. Ha, D. Chun, J.H. Bae, K. Yoon, M. Lee, J. Woo, C. Won, S. Lee, *Adv. Funct. Mater.*, **2020**, *30,* 2005447.

[20] Y. Bai, R. Zhang, X. Ye, Z. Zhu, H. Xie, B. Shen, D. Cai, B. Liu, C. Zhang, Z. Jia, *Nat. Nanotechnol.*, **2018**, *13,* 589.

[21] J. Li, A. Celiz, J. Yang, Q. Yang, I. Wamala, W. Whyte, B. Seo, N. Vasilyev, J. Vlassak, Z. Suo, *Science*, **2017**, *357,* 378.

[22] F.B. Kadumudi, M. Hasany, M.K. Pierchala, M. Jahanshahi, N. Taebnia, M. Mehrali, C.F. Mitu, M.A. Shahbazi, T.G. Zsurzsan, A. Knott, *Adv. Mater.*, **2021**, 2100047.

[23] L. Pan, F. Wang, Y. Cheng, W.R. Leow, Y.-W. Zhang, M. Wang, P. Cai, B. Ji, D. Li, X. Chen, *Nat. Commun.*, **2020**, *11,* 1.

[24] J.-E. Park, H.S. Kang, J. Baek, T.H. Park, S. Oh, H. Lee, M. Koo, C. Park, *ACS Nano*, **2019**, *13,* 9122.

[25] X. Zhao, *Proc. Natl. Acad. Sci. U.S.A.*, **2017**, *114,* 8138.

[26] J. Pan, Y.P. Ivanov, W. Zhou, Y. Li, A. Greer, *Nature*, **2020**, *578,* 559.

[27] A. Seyam, A. El-Shiekh, *Text. Res. J.*, **1993**, *63,* 371.

[28] H. Edwards, D. Farwell, *J. Raman Spectrosc.*, **1995**, *26,* 901.

[29] M. Liu, X. Pu, C. Jiang, T. Liu, X. Huang, L. Chen, C. Du, J. Sun, W. Hu, Z.L. Wang, *Adv. Mater.*, **2017**, *29,* 1703700.

[30] R.D.I.G. Dharmasena, K. Jayawardena, C. Mills, J. Deane, J. Anguita, R. Dorey, S. Silva, *Energy Environ. Sci.*, **2017**, *10,* 1801.

[31] S. Niu, Z.L. Wang, *Nano Energy*, **2015**, *14,* 161.

[32] M. Hargreaves, L.L. Spriet, *Nat. Metab.*, **2020**, *2,* 817.

[33] S. Kipp, W.C. Byrnes, R. Kram, *Appl. Physiol. Nutr. Metab.*, **2018**, *43,* 639.

[34] J.B. Sørensen, J. Kragstrup, K. Kjær, L. Puggaard, *BMC Health Serv. Res.*, **2007**, *7,* 1.

[35] A. Jeukendrup, G. Wallis, *Int. J. Sports Med.*, **2005**, *26,* S28.

[36] P.H. Peckham, J.S. Knutson, *Annu. Rev. Biomed. Eng.*, **2005**, *7,* 327.

[37] Y. Koizumi, S. Iwami, *Theor. Biol. Med. Model.*, **2014**, *11,* 1.

[38] G.A. Gaesser, D.C. Poole, *Exerc. Sport Sci. Rev.*, **1996**, *24,* 35.

References of Figure 4c

[1] L. Pan, F. Wang, Y. Cheng, W.R. Leow, Y.-W. Zhang, M. Wang, P. Cai, B. Ji, D. Li, X. Chen, *Nat. Commun.*, **2020**, *11,* 1.

[2] Z. Xu, Z. Liu, H. Sun, C. Gao, *Adv. Mater.*, **2013**, *25,* 3249.

[3] S. Zhu, J.H. So, R. Mays, S. Desai, W.R. Barnes, B. Pourdeyhimi, M.D. Dickey, *Adv. Funct. Mater.*, **2013**, *23,* 2308.

[4] J.J. Vilatela, R. Marcilla, *Chemistry of Materials*, **2015**, *27,* 6901.

[5] M.K. Shin, J. Oh, M. Lima, M.E. Kozlov, S.J. Kim, R.H. Baughman, *Adv. Mater.*, **2010**, *22,* 2663.

[6] A.J. Granero, P. Wagner, K. Wagner, J.M. Razal, G.G. Wallace, M. in het Panhuis, *Adv. Funct. Mater.*, **2011**, *21,* 955.

[7] F. Xu, Y. Zhu, *Adv. Mater.*, **2012**, *24,* 5117.

[8] M. Park, J. Im, M. Shin, Y. Min, J. Park, H. Cho, S. Park, M.-B. Shim, S. Jeon, D.-Y. Chung, *Nat. Nanotechnol.*, **2012**, *7,* 803.

[9] Y.Y. Huang, E.M. Terentjev, *Adv. Funct. Mater.*, **2010**, *20,* 4062.

[10] J.Y. Oh, S. Kim, H.K. Baik, U. Jeong, *Adv. Mater.*, **2016**, *28,* 4455.

[11] W. Cao, L. Yang, X. Qi, Y. Hou, J. Zhu, M. Yang, *Adv. Funct. Mater.*, **2017**, *27,* 1701061.

[12] R. Templin, R. Sturm, *Journal of the Aeronautical Sciences*, **1940**, *7,* 189.

[13] Y. Wang, C. Zhu, R. Pfattner, H. Yan, L. Jin, S. Chen, F. Molina-Lopez, F. Lissel, J. Liu, N.I. Rabiah, *Sci. Adv.*, **2017**, *3,* e1602076.

[14] S. Liu, K. Li, I. Hussain, O. Oderinde, F. Yao, J. Zhang, G. Fu, *Chemistry–A European Journal*, **2018**, *24,* 6632.

[15] J. Liu, C.S.Y. Tan, Z. Yu, N. Li, C. Abell, O.A. Scherman, *Adv. Mater.*, **2017**, *29,* 1605325.

[16] Z. Wang, J. Chen, Y. Cong, H. Zhang, T. Xu, L. Nie, J. Fu, *Chemistry of Materials*, **2018**, *30,* 8062.

[17] Q. Zhang, X. Liu, L. Duan, G. Gao, *Chemical Engineering Journal*, **2019**, *365,* 10.

[18] S. Naficy, J.M. Razal, G.M. Spinks, G.G. Wallace, P.G. Whitten, *Chemistry of Materials*, **2012**, *24,* 3425.

[19] L. Han, X. Lu, M. Wang, D. Gan, W. Deng, K. Wang, L. Fang, K. Liu, C.W. Chan, Y. Tang, *Small*, **2017**, *13,* 1601916.

[20] F.B. Kadumudi, M. Hasany, M.K. Pierchala, M. Jahanshahi, N. Taebnia, M. Mehrali, C.F. Mitu, M.A. Shahbazi, T.G. Zsurzsan, A. Knott, *Adv. Mater.*, **2021**, 2100047.

[21] Y. Cui, F. Zhang, G. Chen, L. Yao, N. Zhang, Z. Liu, Q. Li, F. Zhang, Z. Cui, K. Zhang, *Adv. Mater.*, **2021**, 2100221.
